# Bidirectional crosstalk between the catalytic and moonlighting functions of human dipeptidyl peptidase 3: potential physiological implications

**DOI:** 10.1039/d6ra05272j

**Published:** 2026-09-14

**Authors:** Antonia Matić, Filip Šupljika, Luka Petohleb, Ana Tomašić Paić, Domagoj C. Kindl, Gordan Horvat, Lea Barbarić, Marina Oskomić, Mihaela Matovina, Antonija Tomić

**Affiliations:** a Ruđer Bošković Institute, Division of Organic Chemistry and Biochemistry Bijenička cesta 54 10000 Zagreb Croatia Antonija.Tomic@irb.hr; b Faculty of Food Technology and Biotechnology, Department of Chemistry and Biochemistry Pierottijeva 6 10000 Zagreb Croatia; c Faculty of Science, Department of Chemistry Horvatovac 102A 10000 Zagreb Croatia

## Abstract

Dipeptidyl peptidase 3 (DPP3) is a ubiquitously expressed zinc-exopeptidase involved in oligopeptide degradation. Beyond its catalytic role, DPP3 exhibits a moonlighting role in the Keap1–Nrf2 signalling pathway, where it promotes Nrf2-dependent gene transcription through an ETGE motif-mediated interaction with the Kelch domain of Keap1. This highlights its role in oxidative stress and cancer, as persistent pathway activation supports tumour cell survival, oxidative stress resistance, and proliferation. While DPP3's catalytic activity is not required for Keap1 binding, the effects of enzyme inactivation on this interaction, and *vice versa*, remain unclear. Using isothermal titration calorimetry and molecular dynamics simulations, we show that DPP3 inactivation, *via* mutation (E451A) or inhibitor binding, enhances DPP3–Keap1 complex formation by facilitating detachment of the ETGE-loop in the closed enzyme conformation, a rate-limiting step in complex assembly. Kinetic measurements further reveal that Keap1 binding modulates DPP3 catalytic activity, increasing efficiency through disproportional reductions in both *k*_cat_ and *K*_m_, likely by stabilizing the closed, catalytically competent conformation of the enzyme, which favours substrate binding but hinders the turnover of reaction participants. These findings reveal a bidirectional regulatory mechanism: catalytic inhibitor binding promotes moonlighting interactions with Keap1, while Keap1 binding enhances enzymatic activity. This dual regulation provides insight into how modulation of DPP3's catalytic function could influence Keap1–Nrf2 redox signaling and suggests how cancer cells might exploit multifunctional enzymes like DPP3 to support tumour progression. This hypothesis based on biochemical and computational data remains to be validated in *in vitro* models.

## Abstract 2

This interdisciplinary study provides evidence for possible communication between DPP3 catalytic activity and its moonlighting function mediated through Kelch domain interaction.

## Graphical abstract

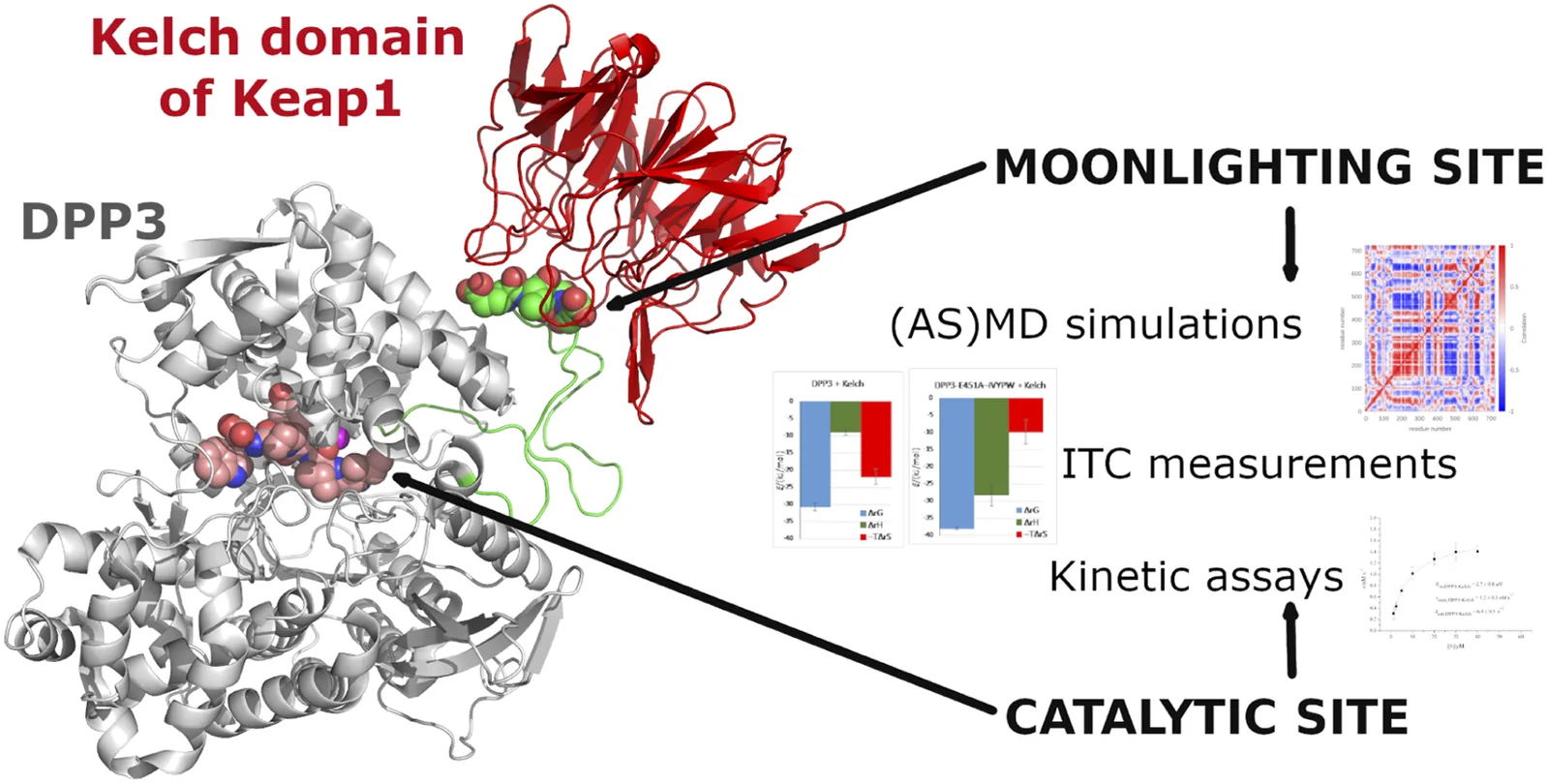

## 1. Introduction

Dipeptidyl peptidase 3 (DPP3) is a metalloprotease that degrades oligopeptides, typically 3–8 amino acids long, by removing dipeptides from their N-terminus.^1^ It is a monomeric protein composed of two domains separated by a wide cleft, with the catalytically active zinc ion located at the base of the upper domain. Experimental and computational studies have shown that DPP3 undergoes conformational changes driven by interdomain movement, with the closed conformation predominating in complexes (pdb_00005e3a, pdb_00005e3c, pdb_00003t6b *etc.*), whereas unbound enzyme structures have been observed in both open (pdb_00003fvy) and closed forms (pdb_00005egy).^2–5^ Computational analyses further indicate the existence of intermediate, so-called semi-closed structures that likely represent the reactive states for substrate binding.^4^ Additionally, these studies suggest that DPP3's conformational change is driven by enthalpy−entropy compensation: the enthalpic increase associated with protein closure is offset by an entropic gain due to the release of structured water molecules from the interdomain cleft into the bulk solvent.^5^ Bezerra *et al.* experimentally demonstrated that substrate binding to the wide-open cleft between the two domains of DPP3 induces conformational changes in the hinge region, driving domain closure.^3^ This motion positions the catalytic zinc ion closer to the carbonyl group of the scissile amide bond. The binding process is primarily entropically driven, mainly due to the release of structured water molecules from the interdomain cleft, while the enthalpic contribution to the binding free energy is unfavourable. Overall, it is postulated that a dynamic equilibrium exists between different DPP3 conformations in aqueous solution. Peptide binding preferentially occurs in the more compact, semi-closed conformation, triggering the final step of active site closure.^3–5^ This supports a mechanism combining both the pre-existing equilibrium and the induced-fit model, which may explain the broad substrate specificity of human DPP3 for peptides of varying lengths and compositions.

*In vitro* studies have shown that DPP3 has a strong affinity for various bioactive peptides, including angiotensins, enkephalins, endomorphins, dynorphins, as well as some hemorphins and exorphins.^6,7^ This substrate promiscuity enables its involvement in numerous physiological processes, including protein catabolism, blood pressure regulation,^8–10^ and pain modulation.^11,12^ Increased expression and activity of DPP3 have been observed in several different types of malignancies. Studies show that the enzyme's amount is significantly elevated in aggressive tumours, such as esophageal,^13^ ovarian,^14^ and breast cancers,^15^ where it promotes tumour cell growth and migration.

**Fig. 1:**
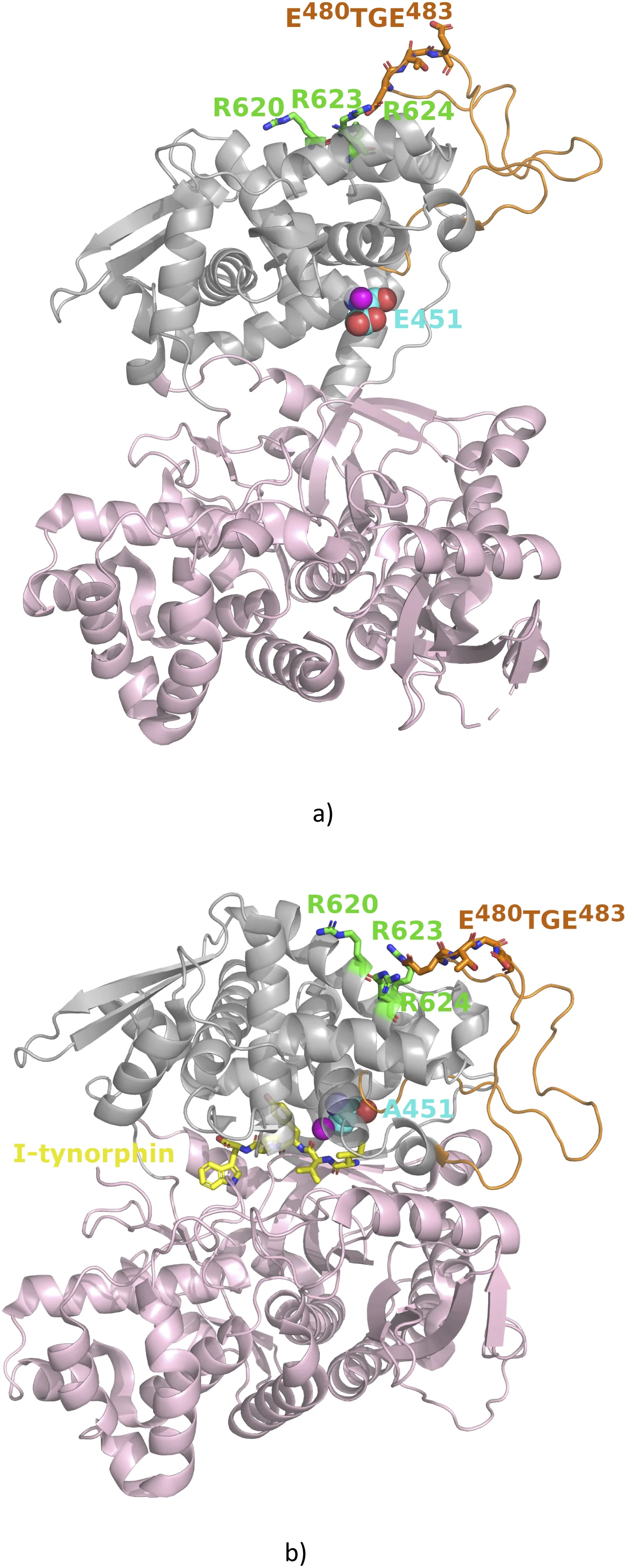
Structures of DPP3 in the (a) open (ligand-free) and (b) closed (in complex with isoleucine derivative of tynorphin; I-tynorphin or IVYPW) conformations, obtained from the Protein Data Bank (pdb_00003fvy and pdb_00005e3c, respectively). The protein lower domain (D3–N336, S375–Q420 and R669–W726) is shown in light pink, and the upper domain (K337–T374 and R421–S668) in light grey, with the ETGE-loop (G454–T494) highlighted in orange. Key residues from the ETGE motif (carbons coloured orange), proximal arginines (carbons coloured green), and I-tynorphin (carbons coloured yellow) are shown in stick representation. The zinc ion (magenta) and the catalytically important mutated residue (carbons coloured cyan) are shown as van der Waals spheres.

In 2013, Hast and colleagues identified DPP3 as one of the proteins in the Kelch-like ECH-associated protein 1 (Keap1) interaction network.^16^ Keap1 protein is a key regulator of the cellular response to oxidative and electrophilic stress, exerting its effects through interaction with the nuclear factor erythroid 2-related factor 2 (Nrf2).^17–20^ Nrf2 is a critical transcription factor that regulates the expression of antioxidant and cytoprotective genes, protecting cells from oxidative stress and damage.^17,21,22^ Under normal conditions, two Kelch domains of Keap1 bind to the ETGE and DLG motifs of Nrf2 in the cytoplasm,^23–25^ promoting its ubiquitination and subsequent proteasomal degradation, thereby maintaining low intracellular Nrf2 levels (Scheme S1, left).^26^ In the presence of oxidative stress or electrophiles, key cysteine residues in Keap1 are modified, triggering conformational changes in the Keap1–Nrf2 complex.^27–29^ These changes prevent the ubiquitination of Nrf2, allowing its accumulation and translocation to the nucleus (Scheme S1, middle) where it activates the transcription of genes coding for proteins involved in antioxidant defence, detoxification, and cellular redox homeostasis.^30,31^ Dysfunction of the Keap1–Nrf2 signalling pathway is linked to various pathological conditions, including cancer^32–37^ and non-cancerous diseases like neurodegenerative disorders (Alzheimer's, Parkinson's, multiple sclerosis),^38^ cardiovascular diseases^39^ and diabetes.^40^ Mutations in the KEAP1 gene that reduce protein functionality or enhance Nrf2 activity can result in persistent Nrf2 activation.^41^ This enables tumour cells to evade oxidative stress, resist apoptosis, and enhance proliferation.^26^ Targeting this pathway opens opportunities for treating various oxidative stress-related diseases, including neurodegenerative disorders, cardiovascular conditions, and cancer. Human DPP3 directly interacts with the Kelch domain of the Keap1 *via* its E^480^TGE^483^ amino acid motif, located on the unstructured loop of the upper domain (further in text referred as ETGE-loop, coloured orange in Fig. 1),^42,43^ competing with the lower-affinity DLG site of Nrf2 for Keap1 binding, thereby inhibiting Nrf2 ubiquitination and promoting Nrf2-dependent transcription (Scheme S1, right).^16,24,44^

Although numerous crystal structures of DPP3 and the Keap1 Kelch domain have been determined, the only available experimental structural information on their complex comes from small-angle X-ray scattering (SAXS) measurement.^42^ The SAXS data for the DPP3–Kelch complex are consistent with computationally predicted models, which indicate that the binding interface is primarily formed between the ETGE-loop of DPP3 and the pore located on the bottom side of the six-bladed β-propeller Kelch domain, surrounded by the B–C and D–A loops.^42,43^ AlphaFold3 predicts a relative orientation of DPP3 and the Kelch domain that is in good agreement with the model obtained by molecular modelling (Fig. S1). However, the predicted models have lower interface predicted template modelling (ipTM) scores (<0.2) than predicted template modelling (pTM) scores (>0.6) (Fig. S1), suggesting that AlphaFold3 has greater confidence in the overall DPP3–Kelch orientation than in the precise protein–protein interface and its specific contacts. This lower confidence may be related, at least in part, to the ETGE-loop conformational rearrangement required for interaction with the Kelch domain, as discussed further in the text. Most predicted complexes feature DPP3 in the closed conformation. In addition, AlphaFold3 predicts several alternative orientations of the Kelch domain relative to DPP3. These models differ primarily in the rotational position of the Kelch domain about the six-fold axis of its β-propeller relative to DPP3. Because all predicted orientations exhibit similar confidence metrics, the results do not support a preferred rotational orientation of the Kelch domain with respect to DPP3, nor do they indicate a significant effect of the E451A mutation on complex formation.

Combining results from experimental and computational study, Matić *et al.* (2021) determined that the binding between DPP3 and Keap1 occurs in two steps: the first step involves an endergonic detachment of the ETGE-loop from the protein upper domain, while the second step involves an exergonic interaction between DPP3 and the Kelch domain.^42^ Hast *et al.* demonstrated that deletion of the ETGE motif in DPP3 abolishes binding to both full-length Keap1 and its Kelch domain.^16^ Importantly, they also confirmed interactions between endogenous DPP3 and Keap1. Furthermore, both stably expressed wild-type (WT) and the catalytically inactive Y318F mutant of DPP3 bound endogenous Keap1, indicating that catalytic activity is not required for this interaction.

In the X-ray structure of DPP3, the ETGE-loop is firmly attached to the protein body, establishing robust salt bridge interactions with adjacent arginines (primarily R623 and R624, but also R620, see Fig. 1),^2,3,6^ a phenomenon observed in molecular dynamic (MD) simulations as well.^42^ In 2022, our group showed that the R623W variant of DPP3 upregulates NAD(P)H dehydrogenase (quinone) 1 (NQO1) expression at the transcriptional level, using NQO1 as one of the markers for Nrf2 activation, supporting the hypothesis that the increased affinity of the R623W variant for Keap1 (ref 42) leads to upregulation of the Keap1–Nrf2 pathway.^45^ These findings, together with those of other studies,^14–16,46–48^ offer new insights into the role of DPP3 in cancer progression and prognosis. In general, tumorigenesis appears to be associated with higher DPP3 expression, as suggested by studies on breast,^15,46^ colorectal^47^ and lung^16^ cancer, which are among the most commonly diagnosed malignancies. These studies showed that DPP3 overexpression is correlated with significantly increased expression of genes controlled by Nrf2. Thus, the molecular mechanism of the involvement of DPP3 in the development of breast cancer is based on the overproduction of DPP3, which could directly lead to an increased antioxidant response through competitive binding with Keap1 which prevents Nrf2 ubiquitination, promoting the growth of cancer cells through increased protection from reactive oxygen species (ROS).^49^ Therefore, DPP3 is recognized as a multifunctional protein that, in addition to its catalytic peptidase activity, also exhibits so-called moonlighting activity in regulating the cellular response to oxidative stress.

The aim of this study is to investigate the potential crosstalk between the catalytic and moonlighting functions of DPP3, with a particular focus on elucidating whether and how these activities influence each other. The resulting insights could advance our understanding of the roles of DPP3 in physiological processes and disease progression, especially in the context of the Keap1–Nrf2 signalling pathway. Such knowledge may have significant implications for both basic and translational research of DPP3-related diseases and could contribute to more accurate predictions of treatment-associated adverse effects.

We first examined how catalytic inactivation of DPP3 influences the structural and thermodynamic basis of its interaction with the Kelch domain, using a combined experimental and computational approach. Because the peptide used in the experimental study—an isoleucine derivative of tynorphin (IVYPW)—is in fact a substrate that is slowly hydrolysed by DPP3, we used a catalytically inactive mutant in which the glutamate at position 451 was replaced by alanine (referred to as DPP3-E451A, Fig. S2).^50,51^ This residue is essential for catalysis, as it initiates the reaction by deprotonating the water molecule that attacks the scissile peptide bond.^51^ As a result, this mutation strongly inhibits enzymatic activity compared with other experimentally tested variants. The availability of high-quality X-ray structures of the E451A mutant alongside the WT protein provides valuable insight into the structural effects of this mutation. Although the WT structure (pdb_00003fvy) adopts a less compact conformation, with a wider interdomain gap than the E451A mutant (pdb_00005egy), the low RMSD values for the individual domains (0.618 Å for the upper domain and 0.477 Å for the lower domain) indicate that the mutation does not significantly alter the overall fold of either domain or affect ETGE-loop binding (Fig. S3). The slightly different conformations adopted by some highly flexible, solvent-exposed residues within the ETGE-loop (*e.g.*, E483 in Fig. S3) are likely attributable to differences in their crystal environments rather than to biologically relevant or functionally important interactions (Fig. S4). Together with modelling studies showing that the ligand-free WT enzyme samples multiple conformational states, these findings suggest that the catalytically inactive, ligand-free E451A mutant represents a conformationally restricted system that is predominantly biased toward the closed, catalytically active state while preserving native-like domain structures. Importantly, the RMSD of 0.324 Å between the ligand-free and IVYPW-bound DPP3-E451A structures (pdb_00005e3c), both in the closed conformation, indicates that the two states are highly similar (Fig. S3). The effects of the E451A mutation on the structures of both the ligand-bound and ligand-free enzyme, as well as on ligand binding, were further assessed computationally. The binding affinities and thermodynamic parameters of different DPP3 variants, in ligand-bound and unbound state, interacting with the Kelch domain were determined using isothermal titration calorimetry (ITC). Computational studies were also conducted to assess how protein conformation and ligand binding influence the rate-determining step in the formation of the complex with Kelch. In parallel, by monitoring hydrolysis of a synthetic substrate Arg-Arg-2-naphthylamide (Arg_2_-2NA) by free DPP3 and in complex with the Kelch domain, we investigated how Kelch binding influences the enzyme's catalytic properties. Together, these results provide mechanistic insight into the interplay between DPP3 catalytic activity and its moonlighting function mediated by the interaction with Keap1, including how modulation of one process influences the other, with potential physiological and pathological implications.

## 2. Materials and methods

### 2.1. Experimental methods

#### 2.1.1. Chemicals

Tris buffer (Art.-Nr. 5429.3), LB broth (Art.-Nr. X964.2), ampicillin (Art.-Nr. K029.5), isopropyl-ß-d-thiogalacto-pyranoside (IPTG; Art.-Nr. 2316.3), imidazole (Art.-Nr. 3899.2), sodium dodecylsulphate (SDS; Art.-Nr. 8029.3), acrylamide/bisacrylamide solution (29 : 1) (Art.-Nr. A124.1), *N*,*N*,*N*′,*N*'-tetramethylethylenediamine (TEMED; Art.-Nr. 2367.3), tris(2-carboxyethyl)phosphine (TCEP; Art.-Nr. 2367.3), Rotigarose His/Ni beads (Art.-Nr. 1308.2), methanol (Art.-Nr. P717.2), glycine (Art.-Nr. 3908.2), Tween 20 (Art.-Nr. 9127.1), sodium chloride (Art.-Nr. 3957.1) were aquired from Carl Roth (Germany). Ammonium persulphate (APS; Art.-Nr. A7460), β-mercaptoethanol (Art.-Nr. M7154), DNase I (Art.-Nr. D5025) were acquired from Sigma-Aldrich (USA). Bromphenol blue (Art.-Nr. 8122) by Merck (Germany). For visualization of SDS PAGE gels we used PhastGel Blue R tablets (Art.-Nr. 17051801) (Pharmacia, Sweden). I-tynorphin (sequence: IVYPW, purity >98%) (%) (Art.-Nr. PCM 16886) was acquired from Pepmic Co. (China), while Arg_2_-2NA (Art.-Nr. 4000098.0050) was aquired from Bachem (Switzerland).

#### 2.1.2. Protein expression and purification

For the purpose of protein expression, *E. coli* strain BL21-CodonPlus (DE3)-RIL was transformed with either the pLATE31_hDPP3 plasmid, containing the expression cDNA insert for human DPP3 with C-terminal His-tag, or the pET-15b plasmid, containing the expression cDNA insert for the Kelch domain of the Keap1 protein. Preparation of plasmids was previously described.^52^ Plasmid for the expression of protein variant DPP3-E451A was prepared using QuikChange II XL Site-Directed Mutagenesis Kit (Agilent (California)), following the instructions of the manufacturer. The sequences of the mutagenic primers were:

DPP3_E451A_F GGTGGGCCTGCACGCGCTGCTGGGCCATG

DPP3_E451A_R CATGGCCCAGCAGCGCGTGCAGGCCCACC

Bacteria were grown in LB media supplemented with 100 µg mL^−1^ ampicillin. The expression of proteins was induced by the addition of IPTG to a final concentration of 0.25 mmol L^−1^ for DPP3, or 0.5 mmol L^−1^ for the Kelch domain, and bacteria were grown at 18 °C overnight. All proteins were purified by affinity chromatography on Ni-NTA column. Further purification was performed by gel filtration chromatography on the FPLC AKTA pure 25 L1 protein chromatography system (Cytiva Europe GmbH), on a Superdex S200 16/60 column. Protein concentrations were determined using BioDrop DUO micro-volume spectrophotometer (Biochrom, UK) for measuring protein A280 adjusted by their mass extinction coefficient.

#### 2.1.3. Isothermal titration calorimetry (ITC)

ITC experiments were conducted using a Malvern PEAQ-ITC microcalorimeter (MicroCal, Inc. Northampton, MA, USA) in 20 mM Tris buffer (pH = 8.0) containing 150 mmol dm^−3^ NaCl and 1 mmol dm^−3^ TCEP. For titration, DPP3 protein solution (30–40 µmol dm^−3^) was in the cell (200 µL) and Kelch (350–400 µmol dm^−3^) was in the syringe (40 µL). All experiments were performed under the same conditions: temperature 25.0 °C, reference power 5.0 µW, high feedback, stirring speed 500 rpm, spacing 150–240 s and initial delay 60 s to allow for equilibration. For all experiments, control measurements were performed to correct for the heat of dilution (buffer–buffer, syringe material–buffer, and buffer–cell material). The appropriate control data were subtracted from each binding experiment during analysis. Data analysis was carried out using the manufacturer-supplied MicroCal PEAQ-ITC analysis software. All parameters are reported as average value and standard deviation of three independent measurements.

#### 2.1.4. Enzyme kinetics

The kinetic parameters for the hydrolysis of the synthetic substrate Arg_2_-2NA by free DPP3 or by DPP3 in complex with the Kelch domain were determined at 25 °C and pH 7.4 by initial rate measurements, as previously described.^53^ Briefly, reactions were carried out in 0.5 M Tris-HCl buffer, pH 7.4. For reactions with free DPP3, the enzyme was preincubated for 30 min at room temperature with the appropriate volume of 20 mmol dm^−3^ Tris, 150 mmol dm^−3^ NaCl, 1 mmol dm^−3^ TCEP, pH 8.0 buffer. For DPP3–Kelch reactions, DPP3 was preincubated under the same conditions but in the presence of Kelch in the same buffer. The final concentration of DPP3 in the reaction was 0.0002 µM, while the concentration of Kelch in the DPP3–Kelch reactions was 1.78 µM (corresponding to a molar ratio of DPP3 : Kelch = 1 : 9224). Reactions were initiated by addition of the appropriate volume of Arg_2_-2NA substrate, and the release of 2-naphthylamine upon cleavage of Arg_2_-2NA was monitored using a Varian Cary Eclipse Fluorescence Spectrophotometer (Kinetics Application) (Agilent Technologies, Santa Clara, CA, USA) at an excitation wavelength of 332 nm and an emission wavelength of 420 nm for 60 s. Michaelis–Menten kinetic parameters were calculated by weighted non-linear regression analysis using program Origin 7.0 for Windows.

### 2.2. Computational methods

#### 2.2.1. Model preparation

The structure of the ligand-free human DPP3 in its closed conformation, deposited in the PDB under the code 5EGY, was used as the template structure for preparing the unbound WT enzyme. The structure of human DPP3 in complex with I-tynorphin, deposited under the code 5E3C, was used for preparing the bound DPP3. Prior to the simulations, all mutated residues present in these X-ray structures were converted back to the wild-type sequence (S19C, C207E, A451E, C491S, S519C, and S654C). These mutations were performed using *tleap*, a basic preparation tool for simulations included in the AMBER22 program package. For the purpose of simulating the unbound and bound E451A mutant of DPP3, the corresponding glutamate residue was mutated to alanine in the selected WT structures obtained from MD simulations using also *tleap*.

The initial protonation of the protein was adopted from our previous publications.^5,50^ All Arg and Lys residues were positively charged, while Glu and Asp residues were negatively charged, as expected under physiological conditions. With the exception of H568, which was positively charged, all other histidine residues were neutral. All neutral histidines were protonated at the Nε position, except for H450 and H455, coordinating the zinc ion, and H36 which were protonated at Nδ. Parameterization was performed using the ff19SB force field for both the protein and the IVYPW peptide,^54^ and for the zinc ion, the extended 4-ligand hybrid bonded/non-bonded parameters derived in our previous work,^55^ were used. The complexes were neutralized with Na^+^ ions and solvated in an octahedral box filled with OPC water molecules.^56^ The minimum distance between the solvated complex and the edge of the box was 11 Å. The resulting systems were simulated using periodic boundary conditions, and the electrostatic interactions were calculated using the particle-mesh Ewald method.^57^ During all simulations bonds involving hydrogen atoms were constrained using the SHAKE algorithm.

All subsequent energy minimizations were performed with CPU version of program *sander*, while MD simulations were performed utilizing the CUDA-enabled graphic processing units (GPUs) version of program *pmemd* in the AMBER22 program.

#### 2.2.2. Molecular dynamic (MD) simulations

Prior to the MD simulations, the solvated WT DPP3 structures (in both bound and unbound states) were optimized in two cycles, each consisting of 10 000 steps. In the first cycle, the protein was constrained using a harmonic potential with a force constant of 83.68 kJ mol^−1^ Å^−2^. The optimized structures were then gradually heated from 0 to 300 K over 500 ps (time step, d*t* = 2 fs), while maintaining the same harmonic potential constraint (83.68 kJ mol^−1^ Å^−2^) on the protein. Density equilibration at 300 K (using the *NpT* ensemble) with a time step of 2 fs was performed in three successive steps. During the first 500 ps the protein was restrained by a force constant of 83.68 kJ mol^−1^ Å^−2^. During the next two 1 ns long simulations the same force constant was applied but only on the protein backbone atoms. After equilibration, three independent 1 µs-long MD simulations were performed without restraints, with a different random seed (ig = −1) used for each simulation (designated A, B and C). The pressure was maintained at 1 atm using the Berendsen barostat, and the temperature was controlled at 300 K using the Langevin thermostat, with a time step of 2 fs in the *NpT* ensemble.

The E451A mutant of DPP3 (both ligand-free and IVYPW-bound forms) was generated by applying the *in silico* mutation procedure, as previously described, to the equilibrated WT system structures. To allow adaptation to this modification, the mutated systems also underwent full optimization for 2500 steps, followed by a 500 ns equilibration under *NpT* conditions (d*t* = 1 fs) without constraints, enabling both water molecules and the protein to adjust to the mutation. As with the WT systems, these E451A mutants of DPP3 (both ligand-free and IVYPW-bound) underwent three independent (designated A, B and C) 1 µs-long MD simulations under the same conditions as the WT systems.

#### 2.2.3. Adaptive steered molecular dynamics (ASMD) simulations

Salt bridge interactions between arginine residues (R620, R623, and R624) in the α-helix at the top of the upper protein domain and glutamate residues from the ETGE motif are primarily responsible for the tight binding of the ETGE-loop to the upper protein domain. To calculate the potential of mean force (PMF) for the detachment of the ETGE-loop from the protein body—a rate-determining step in the two-step process of DPP3–Kelch complex formation–the reaction coordinate was defined as the distance between the centre of the Cα atoms in the E^480^TGE^483^ motif and the centre of the Cα atoms in the L622-S630 motif, which is part of the α-helix at the top of the upper protein domain.

Since ASMD simulations are typically performed in the *NVT* ensemble, the density-equilibrated structures of WT DPP3 and E451A mutant were subjected to a 4 ns equilibration in the canonical ensemble, consisting of four consecutive 1 ns MD simulations (d*t* = 2 fs). During this equilibration phase, backbone restraints were gradually reduced from 83.68, 41.84, 20.92, 4.184 to 0 kJ mol^−1^ Å^−2^. The final equilibrated structures of WT DPP3 and E451A mutant, in both bound and unbound states, were used as starting points for ASMD simulations. In ASMD simulations, a force constant of 20.92 kJ mol^−1^ Å^−2^ was applied (different force constants were tested in our previous publication).^51^ The reaction coordinate was divided into segments of 1 Å (each segment representing one stage) with the end-to-end distance increased from ∼15 Å to ∼20 Å. Fifty trajectories were simulated per stage. During ligand-free WT DPP3 ASMD simulations various pulling speeds (0.25, 0.5, and 1 Å ns^−1^) were tested to assess their impact on the PMF profiles and identify the optimal balance between simulation speed and conformational sampling. The pulling speed was adjusted by varying the duration of simulations performed in each stage: 4 ns, 2 ns, and 1 ns. After each stage (change in reaction coordinated), the structure closest to the Jarzynski average was determined and used as the starting point for the next step. The ASMD simulations were performed in the *NVT* ensemble with temperature held constant using Langevin dynamics with a collision frequency of 1 ps^−1^. The pulling velocity that resulted in the lowest energy barrier for ETGE-loop detachment in closed WT DPP3 was applied to determine the PMF profiles for loop detachment in open WT DPP3 and WT DPP3–IVYPW complex, as well as in the closed E451A mutant of DPP3, both in its unbound state and in complex with IVYPW.

#### 2.2.4. Dynamic cross-correlation matrix (DCCM)

In order to investigate differences in the global motions of the four systems, dynamic cross-correlation matrices (DCCMs) were constructed using the final 400 ns of each of the three independent 1 µs MD simulations performed for each system, corresponding to the structurally stable phase identified from the RMSD and *R*_g_ profiles. Correlations between the Cα atoms of all protein residues were analysed over the selected time period using the *matrix correl* keyword implemented in the *cpptraj* module of Amber22:1
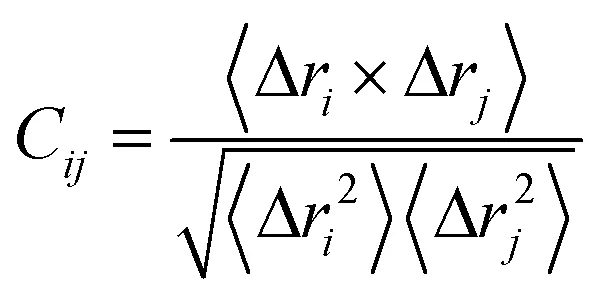
where Δ*r*_*i*_ and Δ*r*_*j*_ represent the displacements of the Cα of the *i*-th and *j*-th protein residues along the analysed trajectory with respect to their positions in the most populated cluster as suggested by M. Castelli *et al.*^58^ The correlation values range from −1 to 1.

## 3. Results and discussion

### 3.1. The catalytically inactive mutant of DPP3 exhibits enhanced affinity for the Kelch domain

**Fig. 2:**
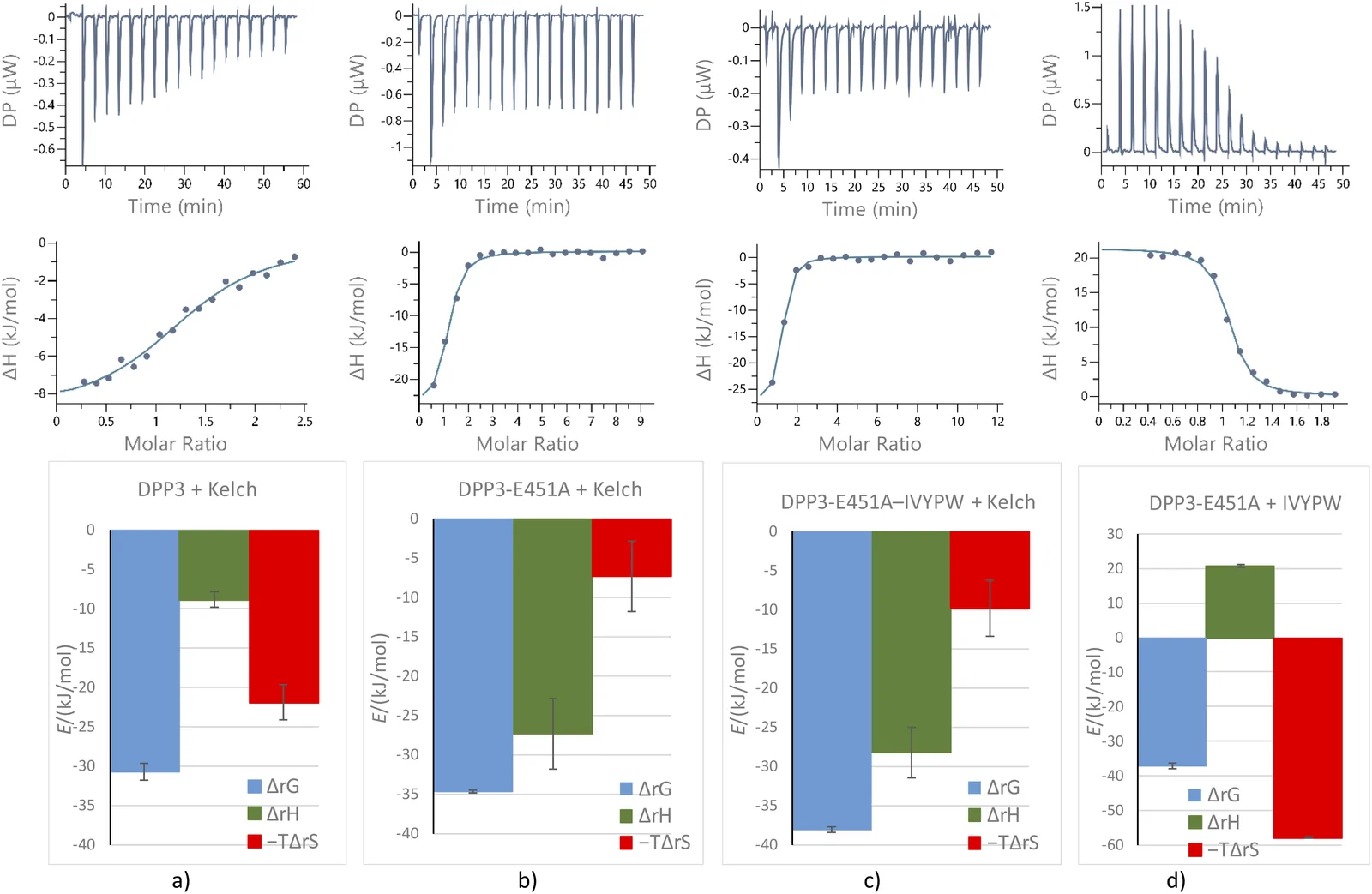
Representative ITC titration curves of: (a) DPP3, (b) DPP3-E451A, and (c) DPP3-E451A–IVYPW complex with Kelch domain, and (d) DPP3-E451A with IVYPW in 20 mmol dm^−3^ Tris buffer, pH = 8.0 with 150 mmol dm^−3^ NaCl and 1 mmol dm^−3^ TCEP at 25.0 °C. Binding isotherms and data with fitting curves are shown.

The interaction between Kelch and WT DPP3 was studied by titrating DPP3 with Kelch in triplicate experiments (see Fig. 2a and S5 and Tables 1 and S1 for details). The obtained ITC results clearly show that a non-covalent interaction between Kelch and DPP3 occurs, and that it is enthalpy and entropy driven. The average dissociation constant (*K*_d_ = 4.52 ± 1.83 µmol dm^−3^) is comparable to the previously reported value of 0.826 µmol dm^−3^ for the WT DPP3–Kelch complex determined by microscale thermophoresis.^42^ In all three titration experiments, the entropic contribution (−*T*Δ_r_*S*) to WT DPP3–Kelch binding was greater than the enthalpic contribution (Δ_r_*H*). The negative −*T*Δ_r_*S* indicates an overall entropy increase upon Kelch binding, likely due to the release of structured water molecules from protein interaction surfaces into the bulk during complex formation, with additional contributions discussed later in text.

**Table 1:** Thermodynamic parameters determined for protein–protein or protein–ligand interactions at 25 °C and pH = 8.0 in 20 mmol dm^−3^ Tris buffer with 150 mmol dm^−3^ NaCl and 1 mmol dm^−3^ TCEP were determined by ITC. The results are mean of three independent experiments with standard deviation. The stoichiometry of the interactions was fixed (*N* = 1) based on previous studies^2,16,42^

Titrand	Titrant	*K*_d_/µM	Δ_r_*G*/kJ mol^−1^	Δ_r_*H*/kJ mol^−1^	−*T*Δ_r_*S*/kJ mol^−1^
DPP3	Kelch	4.52 ± 1.83	−30.7 ± 1.1	−8.9 ± 1.0	−21.9 ± 2.2
DPP3-E451A	Kelch	0.834 ± 0.039	−34.7 ± 0.2	−27.4 ± 4.5	−7.29 ± 4.49
DPP3-E451A–IVYPW	Kelch	0.222 ± 0.039	−38.1 ± 0.4	−28.2 ± 3.2	−9.82 ± 3.53
DPP3-E451A	IVYPW	0.319 ± 0.085	−37.2 ± 0.7	20.8 ± 0.4	−57.9 ± 0.3

Consistent with previous reports, we confirmed that the ETGE motif is essential for interaction with the Keap1 Kelch domain.^16^ Specifically, we investigated the interaction between the Kelch domain and a DPP3 variant lacking four amino acids within the ETGE motif (DPP3–ΔETGE) by titrating DPP3–ΔETGE with Kelch and *vice versa*. Two independent measurements for each titration scheme, clearly showed the absence of a detectable non-covalent interaction between the Kelch domain and DPP3–ΔETGE (Fig. S6).

ITC measurements were also used to determine the binding affinity between the Kelch domain and catalytically inactive E451A mutant of DPP3. The interaction between Kelch and DPP3-E451A was studied in three experiments by titrating the mutated enzyme with Kelch (Fig. S7 and Table S2). Results from Table 1 show that the E451A mutation enhances binding of DPP3 to the Kelch domain by more than 5-fold (*K*_d_ = 4.52 ± 1.83 µmol dm^−3^ for complexation with WT enzyme, compared to *K*_d_ = 0.834 ± 0.039 µmol dm^−3^ for complexation with the DPP3-E451A). Although the interaction of both, WT and DPP3-E451A mutant with the Kelch domain are enthalpy and entropy driven, the relative contributions of these factors differ (Fig. 2a and b and Table 1). Specifically, the entropic contribution is more pronounced for WT DPP3–Kelch complex formation than for binding of the DPP3-E451A mutant to the Kelch domain, whereas the opposite trend is observed for the enthalpic contribution.

### 3.2. I-tynorphin binding at the catalytic site further increases the affinity of DPP3-E451A for the Kelch domain

Before investigating how IVYPW binding at the catalytic site of DPP3 affects the enzyme's interaction with the Kelch domain, it was first necessary to determine whether the ligand itself can bind directly to the Kelch domain. Titration of the Kelch domain with IVYPW (Fig. S8) shows no evidence of non-covalent interaction between IVYPW and Kelch. On the other hand, ITC measurements (Fig. S9 and Table S3) indicate that IVYPW binds non-covalently to DPP3-E451A, with a *K*_d_ value of 0.319 ± 0.085 µmol dm^−3^ (Table 1) in good agreement with the previously determined dissociation constant of *K*_d_ = 0.23 ± 0.02 µmol dm^−3^ reported by Bezerra *et al.*^3^ The binding is entirely entropy-driven (Fig. 2 and S9), which supports a binding mechanism at the interdomain site in which ligand binding displaces structured water molecules from the enzyme interior into the bulk solvent and drives a conformational change from the open to the closed state.

The interaction between the inactive DPP3-E451A–IVYPW complex and the Kelch domain was studied by titrating the complex with Kelch. ITC measurements confirmed that the complex forms a non-covalent interaction with Kelch (Tables 1 and S4 and Fig. 1 and S10). Furthermore, analysis of the dissociation constants (Table 1) reveals that IVYPW binding to the DPP3-E451A mutant increases the affinity of the enzyme for Kelch. Specifically, binding affinity increases nearly 4-fold compared to DPP3-E451A alone and more than 20-fold compared to WT DPP3 alone. This trend is reflected in the decreasing dissociation constant (*K*_d_) and the progressively more negative Gibbs free energy (Δ_r_*G*) for complex formation with Kelch, from WT DPP3 (*K*_d_ = 4.52 ± 1.83 µmol dm^−3^ and Δ_r_*G* = −30.7 ± 1.1 kJ mol^−1^) to DPP3-E451A (*K*_d_ = 0.834 ± 0.039 µmol dm^−3^ and Δ_r_*G* = −34.7 ± 0.2 kJ mol^−1^), and further to the DPP3-E451A–IVYPW complex (*K*_d_ = 0.222 ± 0.039 µmol dm^−3^ and Δ_r_*G* = −38.1 ± 0.4 kJ mol^−1^). These results suggest that catalytic inactivation of DPP3 by mutation and/or inhibitor binding may enhance its moonlighting function within the Keap1–Nrf2 pathway. In addition, thermodynamic signature plots (Fig. 2, S7 and S10, and Table 1) clearly indicate that the binding of the DPP3-E451A–IVYPW complex to the Kelch domain is predominantly driven by enthalpy, similar to what was observed for the DPP3-E451A–Kelch interaction.

### 3.3. Both protein globularity increase and I-tynorphin binding promote ETGE-loop detachment

We used computational approaches to elucidate the molecular mechanisms underlying the increased affinity of DPP3 for the Kelch domain induced by the E451A mutation and IVYPW binding at the catalytic site. In addition to the mutant, the WT protein was included in the computational study to distinguish structural and dynamical effects arising specifically from ligand binding from those introduced by the E451A mutation. Moreover, this approach enabled predictive analysis of the WT DPP3–inhibitor system, which was not directly investigated experimentally.

**Fig. 3:**
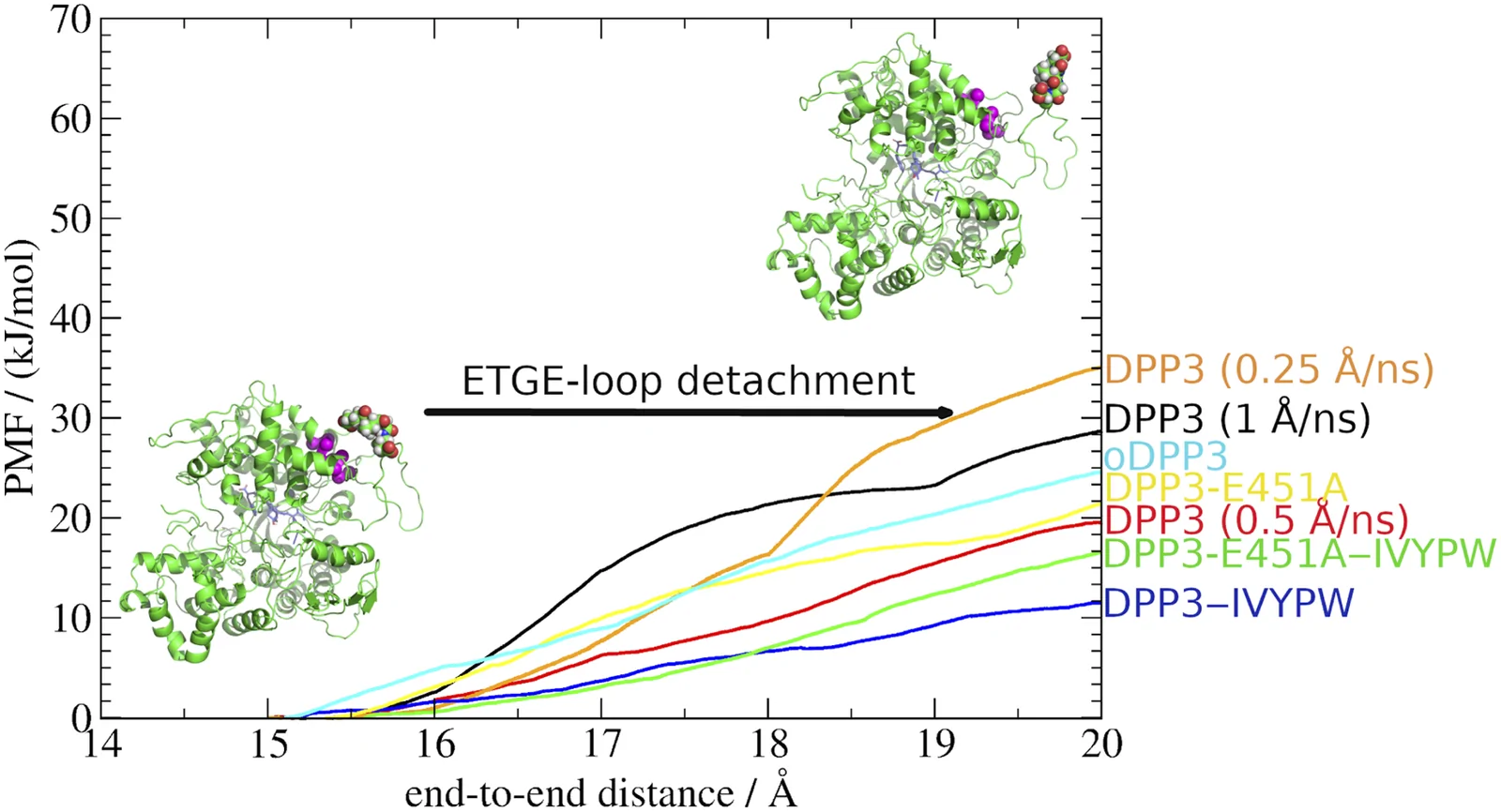
PMF profiles for the detachment of the ETGE-loop from the upper domain of DPP3, obtained from ASMD simulations. The reaction coordinate represents the end-to-end distance between the centres of the Cα atoms of the ETGE motif (depicted as spheres, with carbon atoms in green, oxygens in red, and hydrogens in white) and the Cα atoms of the L622–S630 motif (the Cα atoms shown as spheres and coloured magenta), which is part of the α-helix positioned at the top of the upper protein domain. A force constant of 20.925 kJ mol^−1^ Å^−2^ was applied, and unless indicated otherwise, a pulling velocity of 0.5 Å ns^−1^ was used. I-tynorphin is shown in licorice representation with carbon atom in purple, while the zinc ion is depicted as a grey sphere. The direction of ETGE-loop detachment is indicated by an arrow. If not indicated, WT structures are considered. oDPP3 represents WT ligand-free DPP3 in its open conformation.

Adaptive steered MD (ASMD) simulations were used to gain energetic insight into the initial step of DPP3–Kelch complex formation, which represents the rate-determining step in the binding process. PMF profiles were generated to quantify the energy required for detachment of the ETGE-loop from the upper domain of DPP3 (Fig. 3). To identify the pulling velocity that minimizes the required work and enables estimation of the free energy *via* the Jarzynski equality, we tested several pulling velocities (1.0, 0.5, and 0.25 Å ns^−1^) in the closed, unbound, WT DPP3 system. A pulling velocity of 0.5 Å ns^−1^ yielded the lowest work and the lowest-energy PMF profile (black, red, and orange curves in Fig. 3), and was therefore used in all subsequent ASMD simulations.

According to ASMD results (Fig. 3), the energy required for detachment of the ETGE-loop is highest in the open, ligand-free WT DPP3 (∼25 kJ mol^−1^; cyan line), followed by the closed, unbound WT and E451A mutant DPP3, which exhibit similar energetics (∼20 kJ mol^−1^; red and yellow lines). This indicates that the protein conformational state, rather than the mutation itself, is the primary factor governing the energetics of ETGE-loop detachment in ligand-free structure. It is consistent with the structural analysis presented in the Introduction, which showed that, at the structural level, the E451A mutation primarily affects the protein's tertiary organization by promoting closure without altering domain structure. ASMD simulations further show that IVYPW binding additionally lowers the energy required for loop detachment, with a more pronounced effect in the DPP3–IVYPW complex (∼12 kJ mol^−1^; blue line) than in the DPP3–E451A–IVYPW complex (∼16 kJ mol^−1^; green line). Ligand binding is also known to stabilize the closed conformation od DPP3. However, the difference in ETGE-loop detachment free energies between the ligand-free and ligand-bound closed states suggests that ligand binding influences loop-detachment energetics not only through stabilization of the closed conformation but also through specific ligand–binding-site interactions. Furthermore, the lower free-energy cost of loop detachment observed for the WT DPP3–IVYPW complex compared with the DPP3-E451A–IVYPW complex indicates that the mutational state of the protein may have a greater effect on loop-detachment energetics in the ligand-bound state than in the ligand-free state, likely through specific interactions involving Glu451 and its surrounding residues within the binding site. These results provide submolecular insight into the molecular basis underlying the different binding affinities of the Kelch domain for distinct DPP3 states and their direct consequences for DPP3 moonlighting function.

### 3.4. The E451A mutation and ligand binding stabilize the closed conformation of the enzyme by increasing interdomain contacts

Three independent 1 µs standard MD simulations (designated A, B and C) of WT DPP3 and DPP3-E451A mutant in their closed conformations, both with and without IVYPW, enabled us to evaluate the effect of the mutation and ligand binding on the overall protein structure and dynamic.

The ligand-free enzyme structures exhibit greater structural stability throughout the MD simulations than the IVYPW-bound complexes, as indicated by the RMSD profiles (Fig. S11a). In addition, the E451A mutant appears to form a more stable complex with IVYPW than WT DPP3. Although this difference may partly result from the use of the E451A mutant X-ray structure as a template for constructing the WT DPP3–IVYPW complex, it may also reflect the effect of the mutation itself, as discussed further in Section 3.6. Throughout all simulations of the ligand-free enzymes, DPP3 maintains its closed conformation, as shown by the radius of gyration (*R*_g_) profiles (Fig. S11b) and the final structures obtained after 1 µs of MD simulation (Fig. S12a and c). In contrast, the slight increase in *R*_g_ values (values fluctuate predominantly between 25 and 26 Å) observed during all MD simulations of the DPP3–IVYPW complex and during simulation C of the DPP3-E451A–IVYPW complex is consistent with a slight opening of the enzyme structure. This widening of the substrate-binding site is more pronounced in the WT complex, as indicated by the increased flexibility of several regions of the upper domain (K337–T374, D600–S668, and the ETGE-loop, G454–T494; Fig. S11c) and by the more pronounced increase in the D186–S500 distance during the simulations (Fig. S13). Greater flexibility of the T177-Q191 helix was observed in some of the ligand-free MD simulations (simulation B of DPP3 and simulations A and B of DPP3-E451A; Fig. S11c) compared with the corresponding ligand-bound complexes. The distances shown in Fig. S13 indicate that this increased flexibility results from the movement of this helix slightly farther away from the Y395–Q400 and T494–S500 helices. As expected, apart from the surface-exposed E203–Y211 loop, the greatest flexibility was observed in the ETGE-loop region across all simulations. The highest RMSF values for the ETGE-loop were observed during simulation C of both ligand-free proteins, in which spontaneous ETGE-loop detachment was observed (Fig. S14). Although spontaneous ETGE-loop detachment was not observed during the MD simulations of the ligand-bound complexes, which, according to the ASMD results, exhibit a lower free-energy cost of detachment than the ligand-free states, the predicted detachment free energies (12–25 kJ mol^−1^) indicate that the process is energetically accessible at room temperature, albeit with different probabilities. Therefore, the absence of spontaneous detachment during the unbiased MD simulations of complexes is likely attributable to the stochastic nature of the process and the limited sampling provided by three independent 1 µs simulations, rather than to an energetically prohibitive barrier. In the simulations in which the ETGE-loop remained stably attached to the upper protein domain, its stability was primarily maintained by salt bridge interactions between Glu480, a residue from the ETGE motif, and the nearby arginine residues R620, R623, and R624 located in the α-helix at the top of the upper domain (Fig. S15). Of these interactions, the E480–R624 salt bridge seems to be the most persistent.

**Fig. 4:**
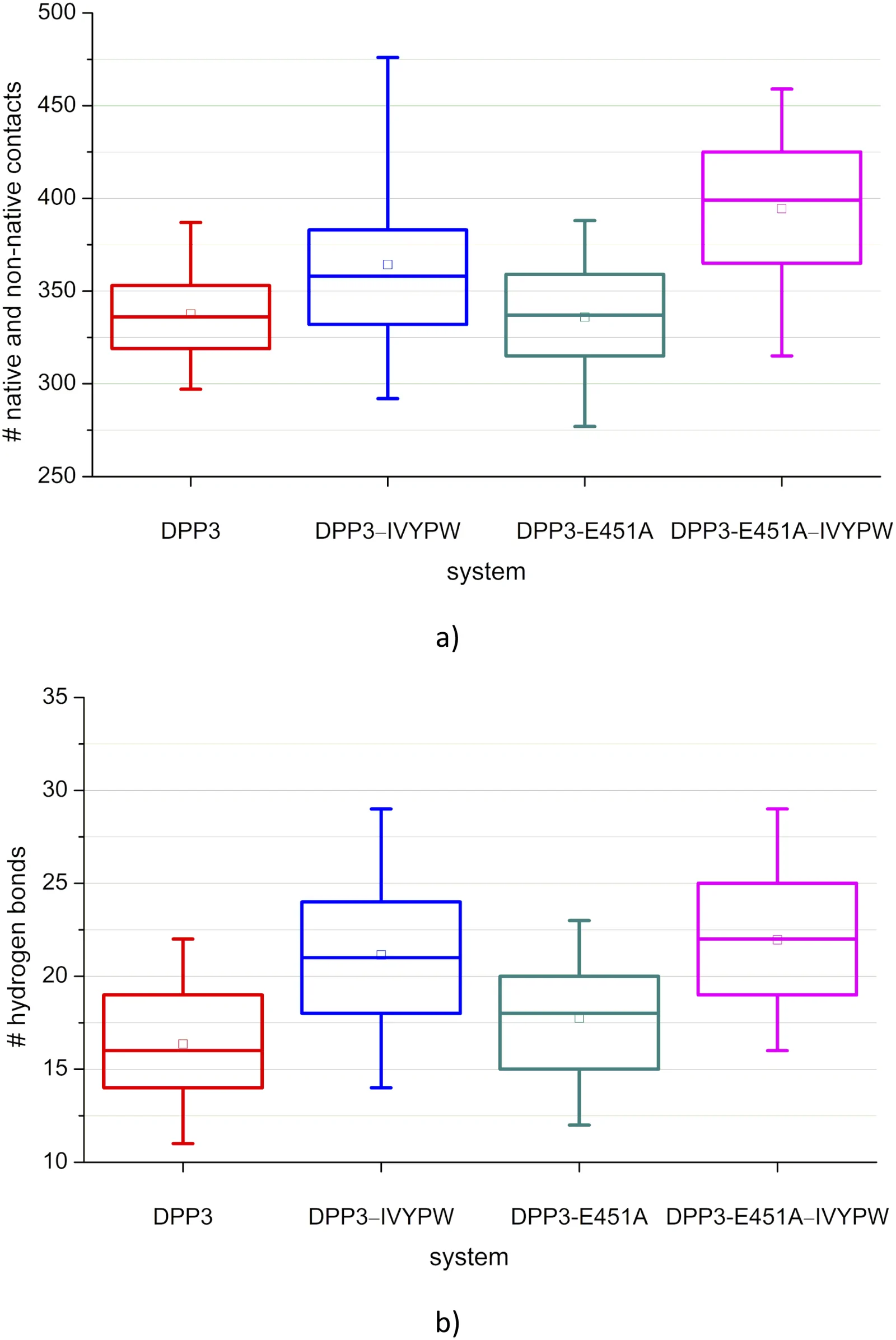
Box plots showing: (a) the number of native and non-native contacts, and (b) the number of hydrogen bonds within DPP3's interdomain cleft. For ligand-free structures, only enzyme residues were considered in the analysis, whereas for ligand-bound complexes, interactions between the ligand and both protein domains were also included. Data were extracted from structures sampled every 400 ps over all 1 µs MD simulations. A hydrogen bond was defined as having a donor–acceptor distance < 3.2 Å and a donor–hydrogen–acceptor angle < 40°.

Although the closed enzyme conformation was largely maintained throughout all simulations, analysis of interdomain contacts revealed that ligand-bound complexes exhibited a higher average number of hydrogen bonds and an increased total number of interdomain contacts (including both native and non-native interactions) between the upper and lower domains compared with ligand-free systems during the production phase (Fig. 4). In these complexes, ligand interactions with both protein domains contribute to the increased contact density within the interdomain cleft. This enhanced interaction network likely stabilizes the closed conformation, consistent with X-ray crystallography data showing a predominance of the closed state in the complexes. In ligand-free simulations, the catalytically inactive DPP3-E451A mutant formed a higher number of hydrogen bonds within the interdomain cleft than WT DPP3, supporting greater stabilization of the closed conformation in the mutant, also in agreement with the corresponding X-ray structures.

In summary, the E451A mutation appears to have a negligible effect on the overall structure and dynamics of ligand-free, closed DPP3, whereas its impact is more pronounced in the IVYPW-bound state. In the presence of IVYPW, the WT enzyme appears to exhibit greater conformational flexibility and a more pronounced tendency toward slight opening than the DPP3-E451A–IVYPW complex.

### 3.5. The ligand binding promotes coordinated local and interdomain dynamics in DPP3

**Fig. 5:**
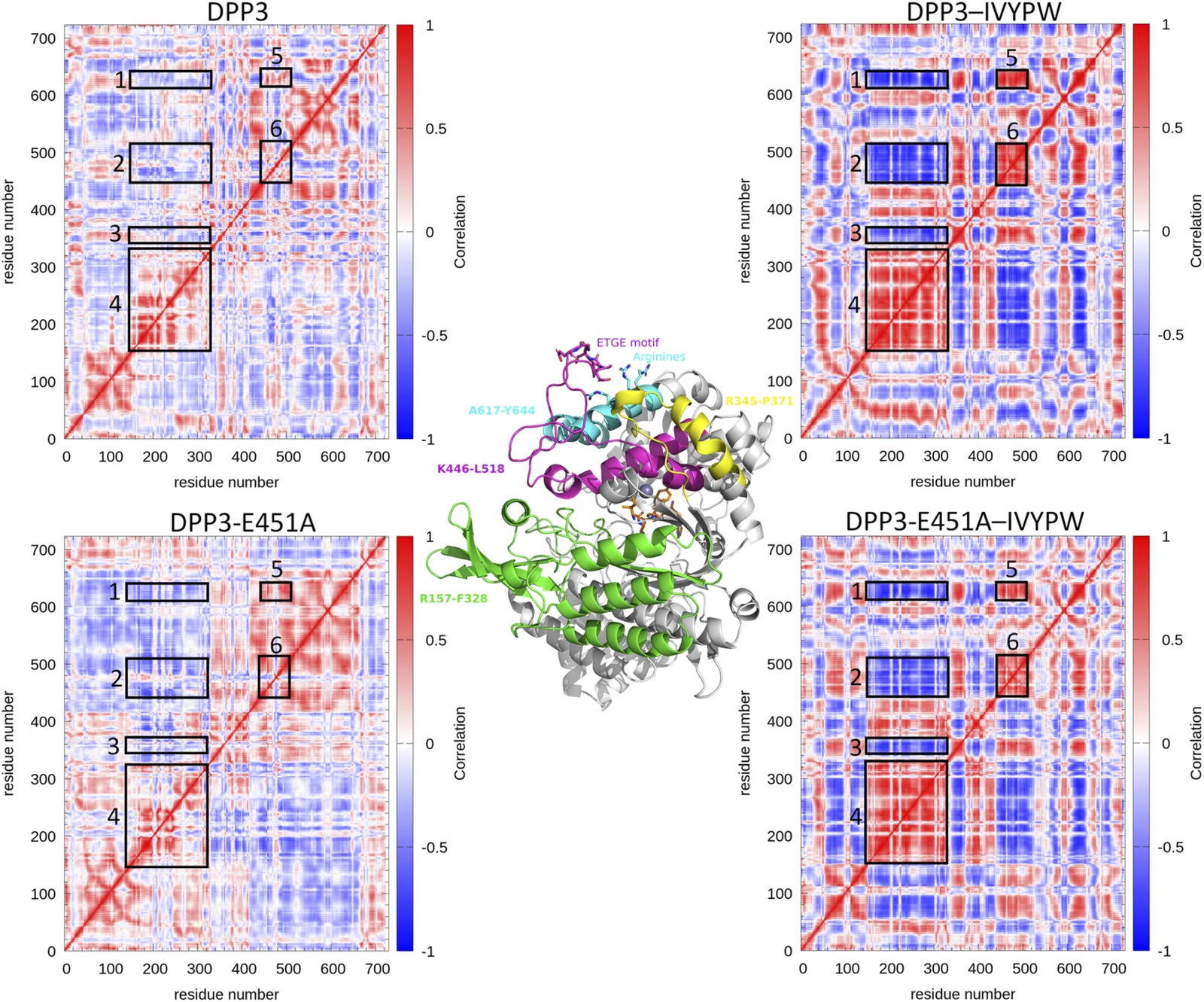
DCCMs of atomic motions over the final 0.4 µs of three independent 1 µs MD simulations for the four systems. Regions of highly correlated and anticorrelated motion are shown in red and blue, respectively. Black rectangles (labelled 1–6) highlight regions that exhibit the most pronounced (anti)correlated motion in the DPP3–IVYPW system. In the middle, regions identified by DCCM analysis: residues R157–F328 (green), R345–P371 (yellow), K446–L518 (magenta), and A617–Y644 (cyan), are show on the structure of DPP3–IVYPW complex (ligand in licorice representation, carbon atoms coloured orange; zinc ion shown as a grey sphere). Amino acids from the ETGE motif and arginine residues that stabilize ETGE-loop binding to the protein surface are shown in licorice representation, with carbon atoms coloured magenta and cyan, respectively.

The degree of coupled motion in the four closed enzyme systems was assessed using dynamic cross-correlation matrices (DCCMs) of atomic fluctuations calculated over the final 0.4 µs of each of the three independent 1 µs MD simulations performed for each system, corresponding to the structurally stable phase indicated by the RMSD and *R*_g_ profiles (Fig. S11a and b). The closed, ligand-free WT system exhibits predominantly weak and spatially dispersed correlations, consistent with limited local and long-range dynamical coupling (Fig. 5). Although regions of enhanced coupled motion (rectangles labelled 1–6 in Fig. 5) begin to emerge in the ligand-free E451A mutant relative to the ligand-free WT structure, these correlations are less pronounced and less uniformly distributed across the highlighted regions than in the ligand-bound systems, likely resulting in a weaker overall effect on DPP3-E451A dynamics. In contrast, ligand binding induces stronger correlated and anticorrelated motions, as well as the formation of larger and more spatially coherent (anti)correlation patterns, with these effects being slightly more pronounced in the DPP3–E451A complex. In particular, IVYPW binding enhances local (near-diagonal) correlated motions within regions of the lower domain (rectangle 4; green region) and the upper domain (rectangle 6; magenta region), as well as between two regions of the upper domain (rectangle 5: residues K446–L518 (magenta) and A617–Y644 (cyan)). Specifically, rectangle 6 indicates pronounced dynamic coupling between residues in the ETGE-loop and residues coordinating the catalytic zinc ion (H450, H455 and E508) and participating in hydrolysis (E451), while rectangle 5 reflects coupling between residues from rectangle 6 and an arginine-rich α-helix in the upper domain that stabilizes ETGE-loop binding to the protein surface. Since the moonlighting function of DPP3 is known to be mediated by the ETGE-loop (G454–T494), which binds the Kelch domain, these findings provide a potential molecular basis for the coupling between catalytic site and DPP3's moonlighting activity, and suggest a possible allosteric communication pathway connecting these two functional sites. At the same time, ligand binding increases anticorrelated motions between region R157–F328 (green) of the lower domain and regions R345–P371 (yellow; rectangle 3), K446–L518 (magenta; rectangle 2) and A617–Y644 (cyan; rectangle 1) of the upper domain. These changes indicate stronger interdomain coupling between distant regions upon ligand binding. Overall, ligand binding is associated with increased dynamical coupling throughout the protein and a more uniform distribution of (anti)correlation patterns across larger protein regions, suggesting more coordinated dynamics and reduced conformational heterogeneity compared to the closed ligand-free states, which may contribute to the reduced ETGE-loop detachment energy observed in the closed ligand-bound states. The increase in dynamic coupling is slightly more pronounced in the DPP3-E451A–IVYPW complex than in the WT complex. In particular, the mutant complex exhibits additional regions of correlated and anticorrelated motion between the lower-domain regions D71–Y95 and N117–A132, which do not directly participate in ligand binding, and the regions coloured green, magenta, cyan, and yellow in Fig. 5. This pattern suggests that the E451A mutation may further enhance interdomain communication in the DPP3–IVYPW complex relative to the WT complex.

### 3.6. The E451A mutation stabilizes the binding-site architecture in the IVYPW-bound complex

Antiparallel binding of peptide ligands to the β-sheet of the lower protein domain is characteristic of DPP3. The positively charged N-terminus of the peptide positions it for this binding mode, placing the scissile peptide bond in proximity to the catalytic zinc ion. Conventional MD simulations of the complexes showed that the E451A mutation does not affect the antiparallel binding of IVYPW (Fig. S16). In simulations of both WT DPP3 and the E451A mutant, IVYPW remained stably bound in a β-sheet conformation antiparallel to the β-sheet of the lower domain, maintaining hydrogen bonding interactions with A388, G389, N391, N394, and E316 (Fig. S16). Consistent with this, the two systems exhibited similar average numbers of hydrogen bonds within the binding site (Fig. S17). The only exception is simulation A of the DPP3-E451A–IVYPW system, in which the ligand retained only hydrogen bonds between its N-terminus and residues N391, N394, and E316 (Fig. S16).

**Fig. 6:**
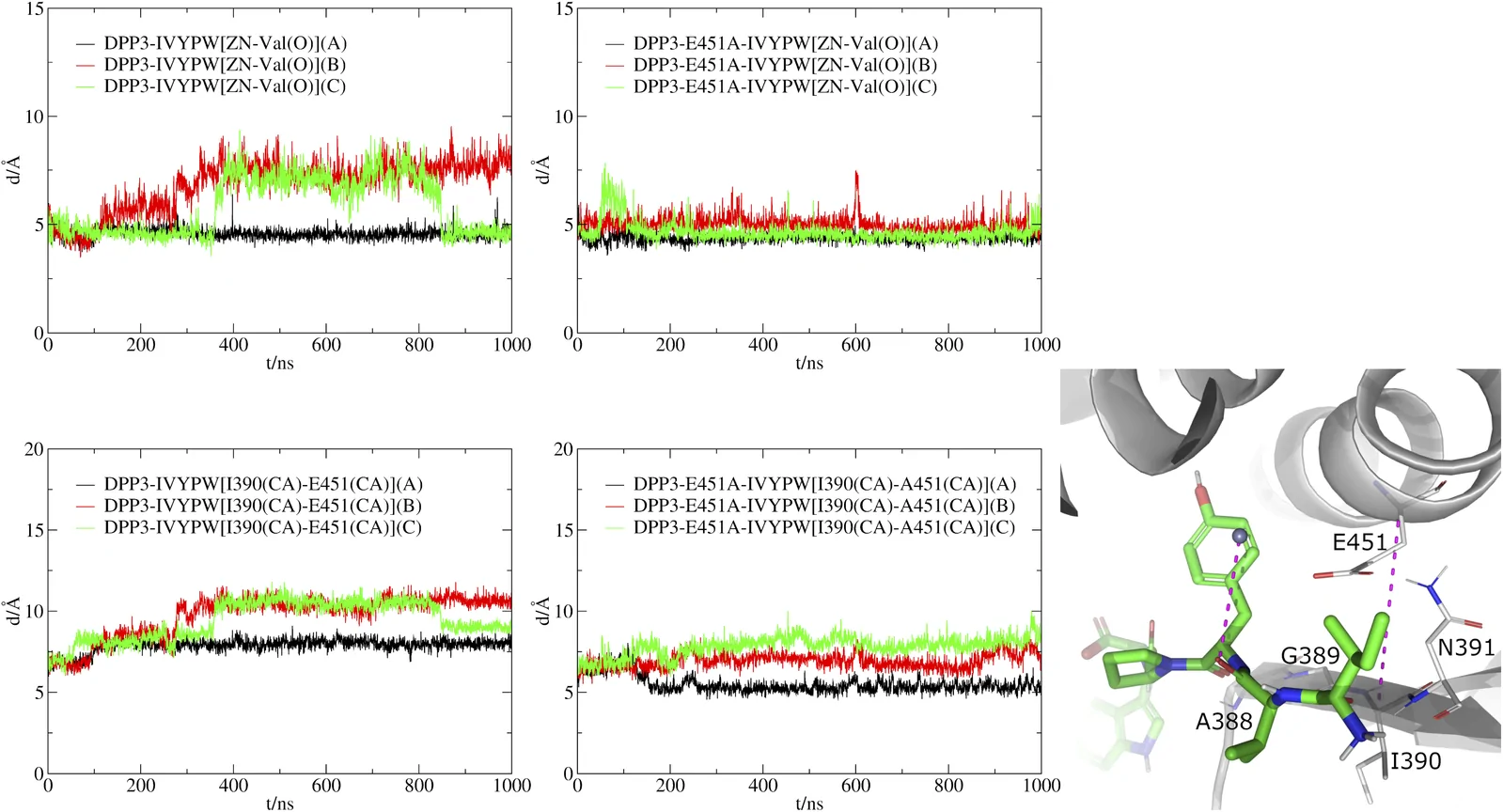
Distance between the catalytic zinc ion and the carbonyl oxygen of the scissile peptide bond, which becomes coordinated to the zinc ion during the hydrolytic reaction (upper row). Widening of the catalytic binding site during three independent MD simulations of the complexes, assessed by the distance between the Cα atoms of I390 in the β-sheet of the lower domain, which participates in antiparallel ligand binding, and residue 451 in the α-helix of the upper domain (E451 in WT and A451 in the E451A mutant) (lower row). The selected distances are indicated by magenta dashed lines on the structure of the DPP3–IVYPW complex.

In contrast, the E451A mutation appears to stabilize the interaction between the catalytic zinc ion and the ligand. More specifically, during the MD simulations, the distance between the zinc ion and the carbonyl oxygen of the scissile peptide bond, which becomes coordinated to the zinc ion during hydrolysis, was more stable in the DPP3-E451A–IVYPW complex than in the WT complex (Fig. 6). This increased stability appears to correlate with changes in the width of the ligand-binding site, as reflected by the distance between the Cα atoms of I390 in the β-sheet of the lower domain, which participates in antiparallel ligand binding, and residue 451 in the α-helix of the upper domain (E451 in WT and A451 in the E451A mutant) (Fig. 6).

Closer examination of the ligand-binding-site architecture during the MD simulations revealed that in the ligand-free structures, which maintained the most compact enzyme conformation, as well as in the DPP3-E451A–IVYPW complex, which exhibited more pronounced structural stability and less pronounced opening-like motion than the WT complex (Fig. S11a,b and S13), a strong hydrogen bond between the side chain of N391 and the carbonyl oxygen of E451/A451 was maintained throughout the simulations (Fig. S18). In contrast, in the DPP3–IVYPW complex, this hydrogen bond was either replaced by an interaction between the N391 side chain and the carboxylate group of E451 (during simulation A, in which the smallest increase in binding-site width was observed, Fig. 6 and S13, and transiently during simulation C) or was completely lost (during simulation B). These observations suggest that the persistent N391–A451 interaction between residues from the two domains may contribute to stabilization of the closed enzyme conformation and, together with additional stabilization provided by ligand binding, may promote enhanced interdomain communication in the DPP3-E451A–IVYPW complex compared with the WT complex.

### 3.7. Kelch preferentially binds DPP3 in its closed conformation

As shown by the thermodynamic signature plots, all DPP3–Kelch interactions are driven by both enthalpic and entropic contributions; however, their relative importance differs among the systems studied (Table 1). Binding of WT DPP3 to Kelch is predominantly entropy-driven, whereas Kelch binding to DPP3-E451A and the DPP3-E451A–IVYPW complex is mainly enthalpy-driven. Although the entropy gain may partly result from the release of ordered water molecules from the protein–protein interface, this effect is expected to be similar across all complexes and therefore cannot fully explain the larger entropic contribution observed for the WT DPP3–Kelch interaction.

Previous experimental and computational studies have shown that ligand-free WT DPP3 adopts multiple conformations in solution. In contrast, our computational results, consistent with X-ray diffraction data, indicate that the E451A mutant preferentially adopts a closed conformation in both ligand-free and ligand-bound states due to an increased number of interdomain contacts. Binding of peptides such as tynorphin (VVYPW) has been reported to be entirely entropy-driven, primarily through the release of structured water molecules from the interdomain cleft, thereby stabilizing the closed conformation of DPP3.^3–5^ Since DPP3-E451A was fully complexed with IVYPW prior to titration with Kelch (Fig. S10), the measured interaction likely involved exclusively the closed conformation of DPP3. The predominance of the closed conformation during titration of unbound DPP3-E451A with Kelch is supported not only by MD simulations and X-ray crystallography data but also by the similar thermodynamic signatures observed for Kelch binding to DPP3-E451A and the DPP3-E451A–IVYPW complex. The approximately fourfold higher dissociation constant for Kelch binding to DPP3-E451A relative to the DPP3-E451A–IVYPW complex may reflect the higher energetic cost of ETGE-loop detachment in the absence of ligand as determined by ASMD simulations (Fig. 3).

Molecular modelling revealed no significant differences in the ETGE-loop detachment energy between the closed ligand-free WT and E451A forms, whereas the open WT conformation exhibited an approximately 2 *k*_B_*T* higher ETGE-loop detachment energy than the closed state (Fig. 3). Combined with evidence that WT enzyme exists in solution as an ensemble of open, semi-closed, and closed conformations, these findings suggest that Kelch preferentially binds the pre-existing closed conformer of WT DPP3. As these conformers become occupied, the equilibrium shifts toward the formation of additional closed-state molecules. This transition is accompanied by the release of ordered water molecules from the DPP3 interdomain cleft, contributing to the favourable entropy change observed for WT DPP3–Kelch complex formation (Fig. S5 and Table 1). In contrast, the E451A mutant, in both the ligand-bound and ligand-free states, predominantly adopts the closed conformation, resulting in a smaller entropic contribution to Kelch binding. This interpretation is supported by previous studies showing that the Kelch complex with closed DPP3 is more stable than that formed with the open conformation, as determined by MM/P(G)BSA analysis,^43^ and by SAXS data demonstrating excellent agreement between experimental envelope and low-energy DPP3–Kelch models containing closed DPP3.^42^ We further validated this by aligning the DPP3 conformations used in the SAXS study with open and closed DPP3 structures from the PDB (Fig. S19).

According to Matić *et al.*,^42^ ETGE-loop detachment is followed by an exergonic interaction between DPP3 and Kelch. Because the catalytic site and the E451A mutation are distant from the protein–protein interface, they are expected to have minimal impact on complex formation once the loop is detached. Accordingly, the free energy gain associated with complex assembly, reflected primarily in the enthalpic contribution, should be similar across all systems. However, the lower exothermicity observed for WT DPP3 binding to Kelch suggests the involvement of additional factors. Previous MD simulations showed that the open-to-closed transition of ligand-free DPP3 is entropically driven, with the unfavourable increase in electrostatic solvation energy compensated by the release of structured water molecules.^5^ Thus, the reduced enthalpic contribution observed for WT DPP3 binding likely reflects the energetic cost of shifting the conformational equilibrium toward the closed state during titration, an effect absent when only the closed conformation enzyme is present.

### 3.8. Catalytic-site inhibitor binding may enhance DPP3's Keap1-mediated moonlighting function

The ITC data clearly demonstrate that both catalytic inactivation of DPP3 by the E451A mutation and I-tynorphin binding at the catalytic site increase the affinity of DPP3 for the Kelch domain. A detailed computational study was therefore undertaken to provide a molecular-level explanation for these effects while also predicting the behaviour of the corresponding WT enzyme–inhibitor complex and assessing whether the trends observed experimentally for the mutant are likely to extend to the WT system.

The computational study showed that, in the closed, ligand-free state of DPP3, the E451A mutation has a negligible effect on protein dynamics (Fig. 5 and S11) and ETGE-loop detachment energetics (Fig. 3), despite a slight increase in the number of interdomain contacts compared with WT DPP3 (Fig. 4). More specifically, ASMD simulations demonstrated that the protein conformational state, rather than the mutation itself, is the primary determinant of ETGE-loop detachment energetics in the ligand-free enzyme, with the free-energy cost of loop detachment being lower in the closed than in the open conformation.

Ligand binding further reduces the free-energy cost of ETGE-loop detachment. This effect can be attributed not only to stabilization of the closed enzyme conformation through an increase in the density of interdomain contacts but also to enhanced coordinated intra- and interdomain motions (Fig. 5) compared to ligand-free states. Although both ligand-bound complexes exhibit lower ETGE-loop detachment free energies than their corresponding closed, ligand-free states, IVYPW binding appears to produce a greater reduction in ETGE-loop detachment free energy in WT DPP3 than in the E451A mutant, with the reduction being approximately 2 *k*_B_*T* greater in the WT complex.

MD simulations further showed that, in addition to the effects of IVYPW binding, the E451A mutation promotes additional correlated and anticorrelated motions in the mutant complex compared with the WT complex. This enhanced interdomain communication in the DPP3-E451A–IVYPW complex may be related to the greater number of interactions formed between the two domains upon ligand binding, particularly the persistent N391–A451 interaction. By strengthening the interdomain contacts, this interaction may contribute to greater stabilization of the binding-site architecture in the E451A complex compared with the WT complex.

The consistency between the trends in ITC-derived binding affinities and ETGE-loop detachment energetics observed for the E451A mutant, both in the absence and presence of IVYPW, supports the predictive relevance of the loop-detachment energetics obtained for the WT DPP3–IVYPW complex. The lower free-energy cost of ETGE-loop detachment, together with the enhanced coordinated dynamics predicted for the WT DPP3–IVYPW complex relative to the ligand-free WT enzyme, suggests that inhibitor binding may strengthen the interaction of WT DPP3 with the Kelch domain, potentially enhancing its moonlighting function in the Keap1–Nrf2 pathway. Moreover, because the predicted loop-detachment free energy is lower for the WT DPP3–IVYPW complex than for the DPP3-E451A–IVYPW complex, the enhancement of Kelch-domain binding upon inhibitor binding may be even more pronounced in the WT enzyme than in the E451A mutant.

### 3.9. Kelch binding increases catalytic efficiency of DPP3

**Fig. 7:**
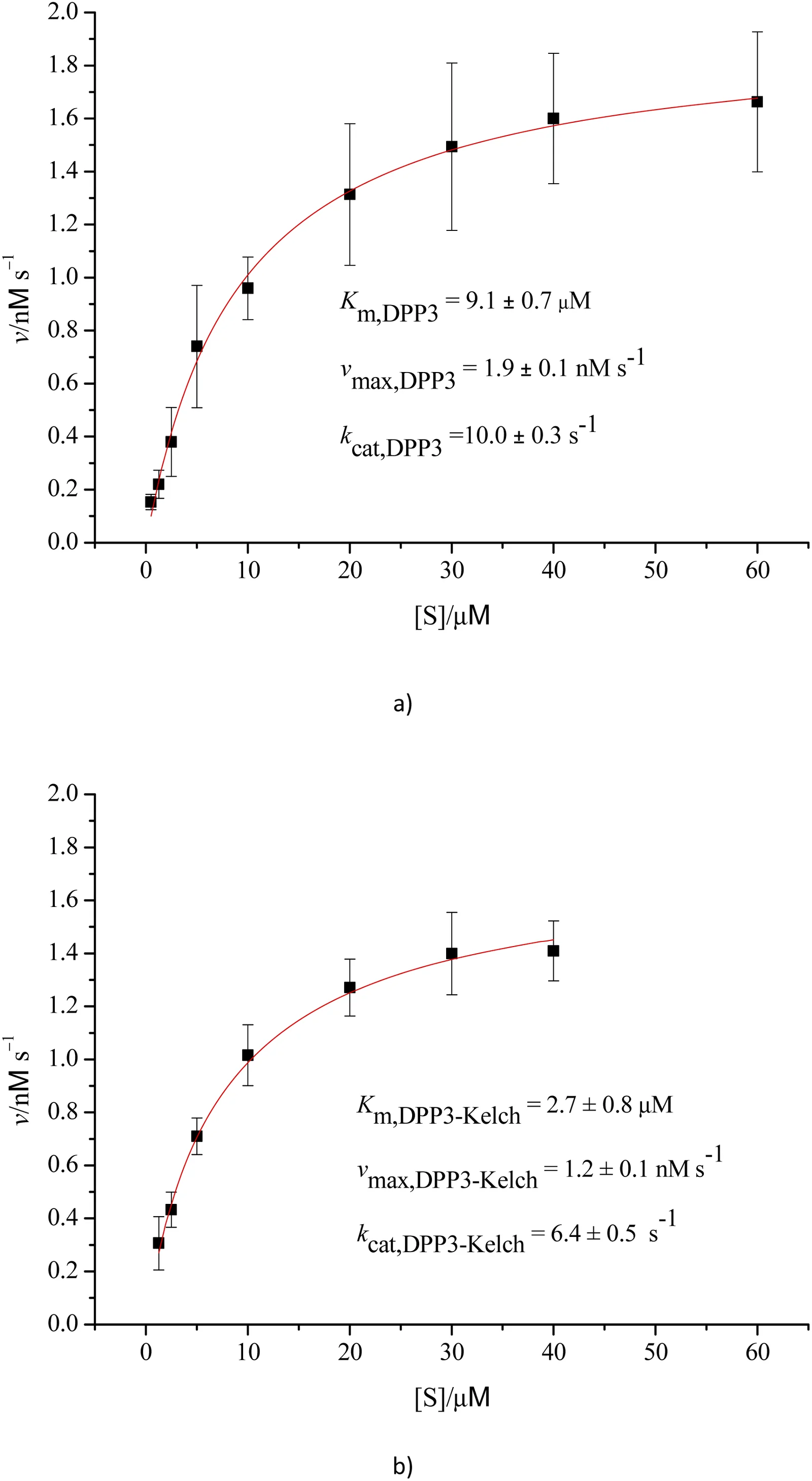
Kinetic analysis of Arg_2_-2NA hydrolysis by: (a) free DPP3 and (b) DPP3–Kelch complex. Kinetic parameters for the DPP3–Kelch complex were derived by applying weighted non-linear regression to the averaged initial velocities from three independent experiments, in which only ∼28% of DPP3 was complexed with Kelch under the experimental conditions.

The preferential binding of Kelch with the closed conformation of DPP3 suggests a potential impact on the enzyme's catalytic efficiency, as the structural plasticity of DPP3 is critical for substrate binding, hydrolysis, and reaction participant trafficking. Previous studies have shown that ligands typically bind to the semi-closed form of DPP3, since the catalytically active closed conformation is too compact to allow ligand transport. To test this hypothesis, we performed kinetic experiments measuring the hydrolysis of Arg_2_-2NA by both free DPP3 and DPP3 preincubated with the Kelch domain (Fig. 7). Cleaving of Arg–Arg from the substrate releases 2-naphthylamine, which was quantified spectrophotofluorometrically to determine kinetic parameters for both the free enzyme and the DPP3–Kelch complex. The first set of parameters was derived from experiments with free DPP3 (Fig. 7a) and the second from the experiment conducted at a DPP3 : Kelch molar ratio of 1 : 9224 (Fig. 7b), which based on the dissociation constant obtained from ITC measurements (*K*_d_ = 4.52 µmol dm^−3^) corresponds to only ∼28% of DPP3 being complexed with Kelch (Fig. S20). To estimate kinetic parameters of substrate hydrolysis at conditions of all DPP3 being in complex with Kelch, weighted non-linear regression analysis was applied on averaged initial velocity values obtained by three independent series of kinetic experiments. In the experiments with DPP3-Kelch mixture, a combined kinetic model of two parallel reactions was used for data fitting:2
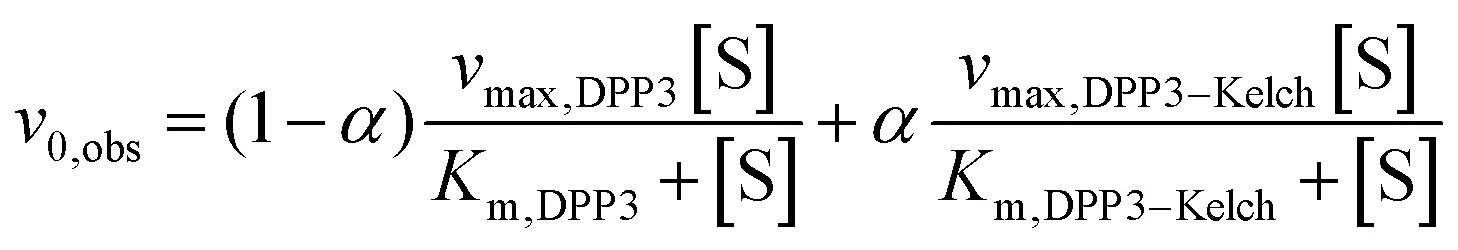
where *α* denotes percentage formation of DPP3–Kelch complex, *v*_max,DPP3_ and *K*_m,DPP3_ are kinetic parameters of free DPP3 substrate hydrolysis, that were kept fixed at the values obtained by separate experiment (Fig. 7a), and *v*_max,DPP3–Kelch_ and *K*_m,DPP3–Kelch_ are kinetic parameters for the hydrolytic activity of DPP3–Kelch complex. In the parallel reaction model, we assumed that substrate binding does not influence the percentage of DPP3–Kelch complex formed and that the lifetime of the DPP3–Kelch complex is significantly longer than the substrate hydrolysis cycle. Although the DPP3–Kelch complex represents only 28% of the total enzyme concentration, its kinetic parameters for Arg_2_-2NA hydrolysis indicate that it is almost saturated in the high-substrate region of the kinetic experiments involving both free and complexed DPP3 (Fig. S21). The complex contributes 22–40% of the overall *v*_0_ values, enabling a reliable kinetic characterization of its hydrolysis reaction.

Overall, the data shown in Fig. 7 indicate that complexation with Kelch resulted in decreases in both *k*_cat_ and *K*_m_ compared to the free enzyme. Specifically, cleavage of Arg_2_-2NA by DPP3–Kelch complex shows a 3.4-fold decrease in *K*_m_ and a 1.6-fold decrease in *k*_cat_ relative to free DPP3. Although Arg_2_-2NA already binds most favourably to the closed conformation of DPP3, the further reduction in *K*_m_ upon Kelch binding indicates that the protein–protein interaction reinforces stabilization of the enzyme–substrate complex, thereby enhancing substrate affinity. However, this enhanced stabilization of the enzyme–substrate complex in the compact enzyme form may slightly hinder substrate access and/or product release, accounting for the concomitant reduction in catalytic turnover (*k*_cat_). Notably, the disproportionate decrease, *K*_m_ decreasing more than *k*_cat_, leads to an approximately twofold increase in catalytic efficiency (*k*_cat_/*K*_m_) for the enzyme when bound to Kelch (*k*_cat_/*K*_m_ = 2.344 × 10^6^ M^−1^ s^−1^) compared to free DPP3 (*k*_cat_/*K*_m_ = 1.099 × 10^6^ M^−1^ s^−1^), indicating that DPP3 becomes more effective at converting substrate to product when bound to Kelch. Recently, epitope mapping studies revealed that Procizumab (PCZ), a first-in-class humanized monoclonal antibody that inhibits DPP3 catalytic activity,^59^ binds to the 7-residue sequence INPETGE.^60^ This represents the first evidence that interactions involving the ETGE motif can modulate DPP3 enzymatic activity, although the underlying mechanism remains unclear.

To assess whether this increase in catalytic efficiency is physiologically relevant, additional analyses were performed. To assess the dose-dependent relationship between hydrolysis rate and Keap1 binding, information on the cellular concentrations of Keap1 and DPP3 is required. The only available data for Keap1 reports a cytoplasmic concentration of ∼1 µM in RAW 264.7 cells,^61^ whereas no absolute intracellular concentrations are available for DPP3. Because Keap1 functions as a homodimer, the effective intracellular concentration of available Kelch domains is roughly twice the Keap1 concentration. Under basal conditions, the abundance of Nrf2, the primary physiological binding partner of Keap1, is approximately threefold lower than that of Keap1, whereas under oxidative stress its concentration nearly doubles relative to Keap1, whose concentration remains unchanged.^61^ Since the binding affinities of the Nrf2 DLG motif (*K*_a_ = (1.0 ± 0.0) *×* 10^6^ M^−1^)^62^ and the DPP3 ETGE motif are comparable, DPP3 can effectively compete with Nrf2 for the DLG-site interaction. This suggests that the concentration range of Kelch binding sites available for DPP3 binding may lie between ∼1.66 and ∼1 µM under basal conditions and oxidative stress conditions, respectively. Fig. S22 illustrates the dependence of the fraction of Kelch binding sites occupied by DPP3 (red and black symbols and lines) and the fraction of DPP3 in complex with Kelch (blue and green symbols and lines) on the DPP3 : Kelch molar ratio. In this analysis, the concentration of Kelch binding sites was fixed at 1.66 µM or 1.00 µM, while the DPP3 concentration was varied. The graph shows that increasing DPP3 concentration beyond that of Kelch progressively increases the fraction of Kelch bound by DPP3, driving the system toward maximal utilization of the catalytic enhancement arising from DPP3–Kelch complex formation. At the same time, however, the fraction of DPP3 in complex with Kelch decreases relative to free DPP3, thereby reducing its contribution to overall hydrolysis. Therefore, if one plots the ratio of hydrolysis rates under physiological conditions where [S] << *K*_m_ (using the equation *v*_0_ = (*k*_cat_/*K*_m_)[E][S]) between a case in which all DPP3 is free (*v*_0,DPP3_) and one in which a fraction of DPP3 is bound to Kelch in addition to the free DPP3 (*v*_0,DPP3_ + *v*_0,DPP3–Kelch_) as a function of the DPP3 : Kelch ratio (Fig. S23), it is evident that the Keap1-induced enhancement of DPP3 catalytic efficiency is the most pronounced when DPP3 concentrations are equal to or lower than those of Kelch, with a contribution ranging from ∼23–20% under basal conditions to ∼17–15% under stress conditions. Increasing the DPP3 concentration to ten times that of Kelch results in an additional contribution of less than 10% to the total hydrolysis rate, and the difference between basal and oxidized states becomes progressively less pronounced. At even higher DPP3 concentrations, both the difference in hydrolysis rates between basal and oxidized states and the contribution of DPP3–Kelch binding to the total hydrolysis rate become negligible. Taken together, these observations suggest that the Keap1-mediated enhancement of DPP3 catalytic efficiency could be physiologically relevant primarily when DPP3 concentrations are comparable to, or up to tenfold higher than, those of Kelch. Nonetheless, conclusive validation of this effect would require further experiments in cell culture model systems, which are beyond the scope of the present study.

Proteolytic enzymes are well known to facilitate cancer invasion, metastasis, and immune evasion by remodelling the extracellular matrix and altering the tumour microenvironment.^63^ Namely, matrix metalloproteinases (MMPs)^64^ and serine proteases (*e.g.*, uPA)^65^ degrade components of the extracellular matrix, allowing cancer cells to break through tissue barriers facilitating invasion and metastasis, while certain tumour-associated peptidases (*e.g.*, dipeptidyl peptidase 4 or DPP4)^66^ can alter the tumour microenvironment, helping cancer cells evade immune surveillance. Potential involvement of DPP3 in malignant transformation was also indicated by the finding that it reduces the immunogenicity of necrotic tumour cells by blocking antigen cross presentation.^67^ Considering these data, the results presented in this study raise the question of whether dual-function DPP3 enzyme could contribute to oncogenic processes through both its catalytic and non-catalytic (moonlighting) activities. Specifically, it is conceivable that the tumour-associated peptidase DPP3 promotes cancer progression by simultaneously leveraging both functions upon binding to Keap1: (i) activation of Nrf2-dependent transcription of cytoprotective genes *via* its moonlighting role, and (ii) enhanced hydrolysis of peptides to provide amino acids that support rapid cellular proliferation and/or reduce tumour immunogenicity.

## 4. Conclusions

This multi-layered study provides, for the first time, submolecular insight into the crosstalk between the catalytic and moonlighting functions of DPP3, a phenomenon previously reported for some metabolic enzymes,^68^ with potential physiological and pathological implications. By integrating experimental approaches (ITC measurements and kinetic analyses) with computational methods (MD and ASMD simulations), we demonstrated that catalytic inactivation of DPP3 enhances its interaction with Keap1. Our results show that both mutation of a catalytically important residue and binding of a peptide increase DPP3 affinity for the Kelch domain by stabilizing the closed enzyme conformation, in which ETGE-loop detachment is energetically more favourable. Although these findings suggest that inhibition of DPP3 catalytic activity may not be an effective strategy for suppressing its involvement in the Keap1–Nrf2 signalling pathway under conditions of DPP3 overexpression, this study provides the first evidence that inhibitor binding at the catalytic site can allosterically modulate the moonlighting function of DPP3, thereby affecting its interaction with Keap1 and, consequently, Nrf2 activity. Importantly, the computational analyses indicate that IVYPW binding reduces the ETGE-loop detachment energy in WT DPP3 more than in the E451A mutant, suggesting that even greater enhancement of Kelch binding may be achieved with more potent DPP3 inhibitors, such as Tyr–Phe–NHOH or JMV-390.^69^

Beyond its established moonlighting role in activating Nrf2-dependent cytoprotective signalling through Keap1 binding, our kinetic analyses demonstrate that interaction with Keap1 enhances the catalytic efficiency of DPP3. This effect can be explained by preferential binding of Kelch to the closed conformation of DPP3, thereby stabilizing the catalytically active state of the enzyme and increasing substrate affinity, as reflected by the lower *K*_m_ value. At the same time, stabilization of the closed conformation likely reduces the conformational flexibility required for efficient catalytic turnover, resulting in a lower *k*_cat_. However, because the reduction in *K*_m_ outweighs the decrease in *k*_cat_, the overall catalytic efficiency increases. This effect may have physiological implications; for example, increased amino acid availability could contribute to rapid cell proliferation and/or reduced tumour immunogenicity.

If confirmed at the cellular level, these finding could suggest how multifunctional enzymes such as DPP3 may be exploited by cancer cells to promote tumour progression. This completes the evolving understanding of metabolic enzyme function, which has evolved from viewing them solely as catalysts of individual metabolic reactions, to recognizing their additional roles in regulation of gene transcription, DNA damage repair, and cell fate decisions, and now to appreciating the interplay between these two functions in supporting pathological processes. These findings also raise the possibility that simultaneous targeting of both the catalytic and moonlighting functions of DPP3 in DPP3-overexpressing tumours could represent an effective strategy for enhancing the sensitivity of malignant cells to anticancer therapies. Although such implications remain speculative and extend beyond the scope of the present study, the results presented here provide a strong foundation for future cellular and *in vitro* investigations of these mechanisms.

## Author contributions

A. M. and F. Š. contributed equally to this work. A. M.: formal analysis, investigation, methodology, validation, writing – original draft. F. Š.: formal analysis, investigation, methodology, validation, visualization, writing – original draft. M. O., L. P., D. C. K. and L. B.: investigation. A. T. P.: investigation, writing – original draft. G. H.: formal analysis, methodology. M. M.: formal analysis, investigation, methodology, resources, supervision, validation, writing – original draft, writing – review & editing. A. T.: conceptualization, data curation, formal analysis, funding acquisition, investigation, methodology, project administration, resources, supervision, validation, visualization, writing – original draft, writing – review & editing.

## Conflicts of interest

There are no conflicts to declare.

## Supplementary Material

RA-OLF-D6RA05272J-s001

## Data Availability

The data supporting this article have been included as part of the supplementary information (SI). Computational data for this article are available at Mendeley data repository at https://data.mendeley.com/datasets/kfv7wb2sc7/1, while experimental data at Fulir data repository at https://urn.nsk.hr/urn:nbn:hr:241:880793. Supplementary information is available. See DOI: https://doi.org/10.1039/d6ra05272j.
