# Supplementary material for: Bidirectional crosstalk between the catalytic and moonlighting functions of human dipeptidyl peptidase 3: potential physiological implications
Source: RSC Adv. 2026 Sep 14. Online ahead of print. doi: 10.1039/d6ra05272j (PMC13573633; doi:10.1039/d6ra05272j)
Supplement: Supplementary file 1 [file RA-OLF-D6RA05272J-s001.pdf]

Supplementary Information (SI) for RSC Advances.
This journal is © The Royal Society of Chemistry 2026

SUPPORTING INFORMATION

Bidirectional crosstalk between the catalytic and moonlighting functions of human dipeptidyl peptidase 3: potential physiological implications

Antonia Matić, Filip Šupljika, Luka Petohleb, Ana Tomašić Paić, Domagoj C. Kindl, Gordan Horvat, Lea Barbarić, Marina Oskomić, Mihaela Matovina, Antonija Tomić

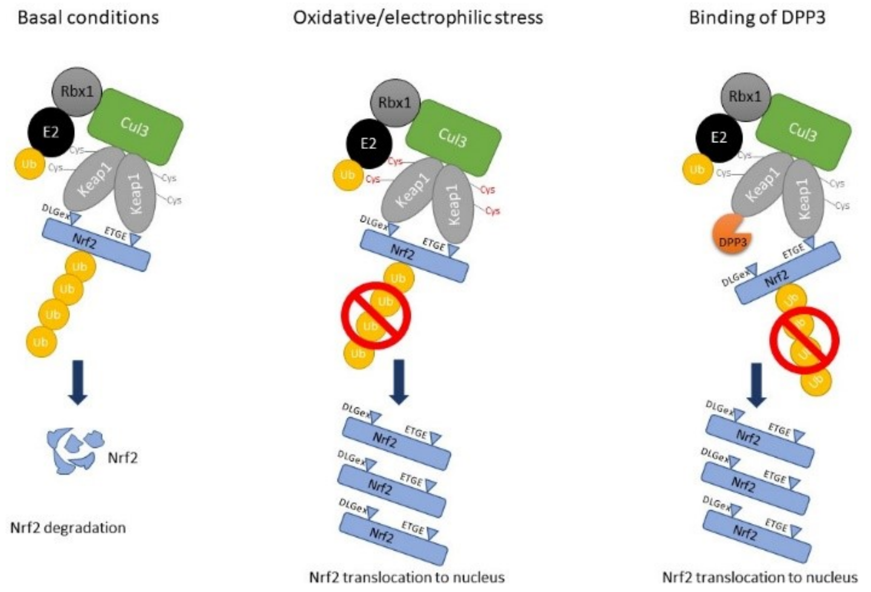

Scheme S1. Schematic overview of the Keap1–Nrf2 signaling pathway. Under basal conditions, Keap1 binds to the ETGE and DLGex motifs on Nrf2 and recruits Nrf2 to the Keap1–Cul3–E3 ubiquitin ligase complex, leading to its ubiquitination and subsequent degradation. Under oxidative stress or in the presence of electrophiles, modification of specific cysteine residues in Keap1 induces conformational changes in the Keap1–Cul3–E3 ligase complex, impairing Nrf2 ubiquitination and degradation. As a result, newly synthesized Nrf2 accumulates and translocates to the nucleus, where it activates the transcription of antioxidative and detoxification genes. Binding of disrupting proteins, such as DPP3, similarly promotes Nrf2-dependent gene transcription by interfering with Keap1 binding to both Nrf2 motifs.

Table S1. ITC titrations of DPP III with Kelch (m_1-m_3), fitting with the model one set of sites. Measurements were done in 20 mM Tris buffer, pH = 8.0 with 150 mM NaCl and 1 mM TCEP at 25.0 °C. The stoichiometry of the interaction (*N*) was fixed to *N* = 1.

measurement	K_d/mol dm^{-3}	$\Delta_r H$/kJ mol^{-1}	$\Delta_r G$/kJ mol^{-1}	$-T\,\Delta_r S$/kJ mol^{-1}
m_1	$2.45 \cdot 10^{-6}$	-7.8	-32.0	-24.3
m_2	$5.18 \cdot 10^{-6}$	-9.0	-30.2	-21.2
m_3	$5.93 \cdot 10^{-6}$	-9.8	-29.9	-20.1

Table S2. ITC titrations of E451A mutant of DPP3 with Kelch (m_1-m_3), fitting with the model one set of sites. Measurements were done in 20 mmol dm^{-3} Tris buffer, pH = 8.0 with 150 mmol dm^{-3} NaCl and 1 mmol dm^{-3} TCEP at 25.0 °C. The stoichiometry of the interaction (*N*) was fixed to *N* = 1.

measurement	K_d/mol dm^{-3}	$\Delta_r H$/kJ mol^{-1}	$\Delta_r G$/kJ mol^{-1}	$-T\,\Delta_r S$/kJ mol^{-1}
m_1	$7.95 \cdot 10^{-7}$	−24.9	−34.9	−9.92
m_2	$8.73 \cdot 10^{-7}$	−24.8	−34.6	−9.85
m_3	$8.34 \cdot 10^{-7}$	−32.6	−34.7	−2.10

Table S3. ITC titrations of E451A mutant of DPP3 with IVYPW (m_1-m_3), fitting with the model one set of sites. Measurements were done in 20 mmol dm^{-3} Tris buffer, pH = 8.0 with 150 mmol dm^{-3} NaCl and 1 mmol dm^{-3} TCEP at 25.0 °C. The stoichiometry of the interaction (*N*) was fixed to *N* = 1.

measurement	K_d/mol dm^{-3}	$\Delta_r H$/kJ mol^{-1}	$\Delta_r G$/kJ mol^{-1}	$-T\Delta_r S$/kJ mol^{-1}
m_1	$2.40 \cdot 10^{-7}$	20.4	−37.8	−58.2
m_2	$4.08 \cdot 10^{-7}$	21.2	−36.5	−57.7
m_3	$3.08 \cdot 10^{-7}$	20.7	−37.2	−57.9

Table S4. ITC titrations of DPP3-E451A–IVYPW complex with Kelch (m_1-m_3), fitting with the model one set of sites. Measurements were done in 20 mmol dm^{-3} Tris buffer, pH = 8.0 with 150 mmol dm^{-3} NaCl and 1 mmol dm^{-3} TCEP at 25.0 °C. at 25.0 °C. The stoichiometry of the interaction (*N*) was fixed to *N* = 1.

measurement	K_d/mol dm^{-3}	$\Delta_r H$/kJ mol^{-1}	$\Delta_r G$/kJ mol^{-1}	$-T\,\Delta_r S$/kJ mol^{-1}
m_1	$2.67 \cdot 10^{-7}$	−31.4	−37.6	−6.16
m_2	$2.01 \cdot 10^{-7}$	−25.1	−38.3	−13.2
m_3	$1.99 \cdot 10^{-7}$	−28.2	−38.3	−10.1

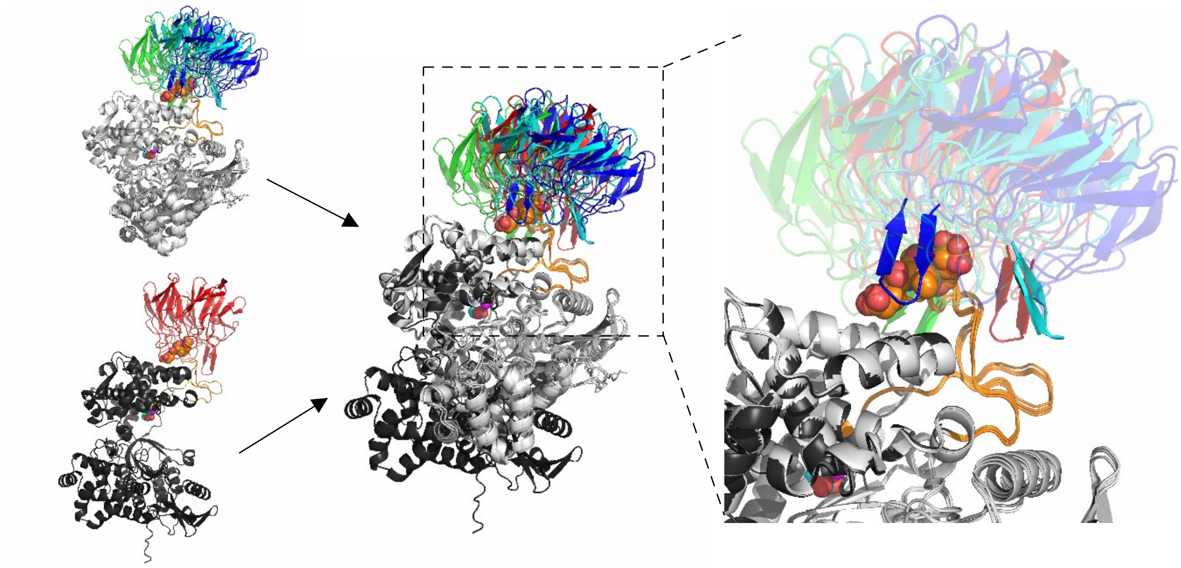

model	ranking score	ipTM	pTM
red	0.26	0.16	0.64
green	0.25	0.16	0.61
blue	0.24	0.15	0.60
cyan	0.25	0.15	0.61

a)

model	ranking score	ipTM	pTM
red	0.24	0.13	0.63
green	0.23	0.13	0.63
blue	0.26	0.16	0.61
yellow	0.25	0.14	0.63
pink	0.24	0.14	0.65

b)

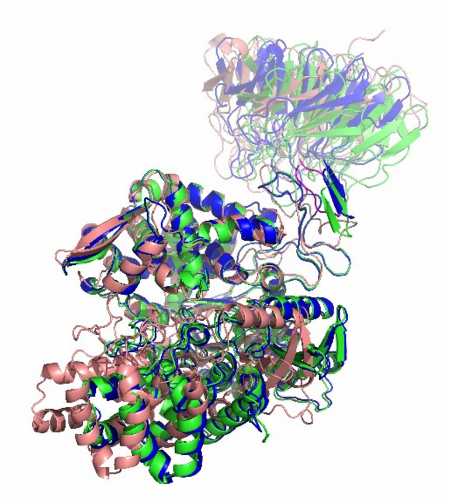

c)

Figure S1. AlphaFold3 (AF3)-predicted models (generated using the default AF3 server parameters; two independent prediction runs, each producing five models) of the complexes formed between (a) WT DPP3 and the Kelch domain and (b) the E451A mutant and the Kelch domain. DPP3 structures in the open and closed conformations are shown in black and light gray, respectively. Herein, only the AF3 models exhibiting a similar orientation of the Kelch domain relative to DPP3 as the molecular modeling structure that is in good agreement with the SAXS experiment, are shown. In the right panels, the Kelch domain is shown as transparent, with different colors corresponding to the individual predicted models, whereas the B–C loop of blade II of the six-bladed β-propeller Kelch domain, which adopts a β-sheet conformation, is shown as opaque. AF3 prediction metrics are provided in the accompanying tables, with model designations corresponding to the colors of the Kelch domains. The zinc ion is represented as a black sphere, while residues of the ETGE-loop are shown in orange, with the ETGE motif heavy atoms highlighted as spheres. (c) Structural alignment of the DPP3–Kelch complex obtained by molecular modeling (pink), which showed good agreement with the SAXS data, with the selected AF3-predicted complexes (WT, green; E451A, blue). The complex structures are aligned according to the DPP3 upper domain. The colors of the predicted complexes correspond to the Kelch model designations in the tables shown in panels (a) and (b).

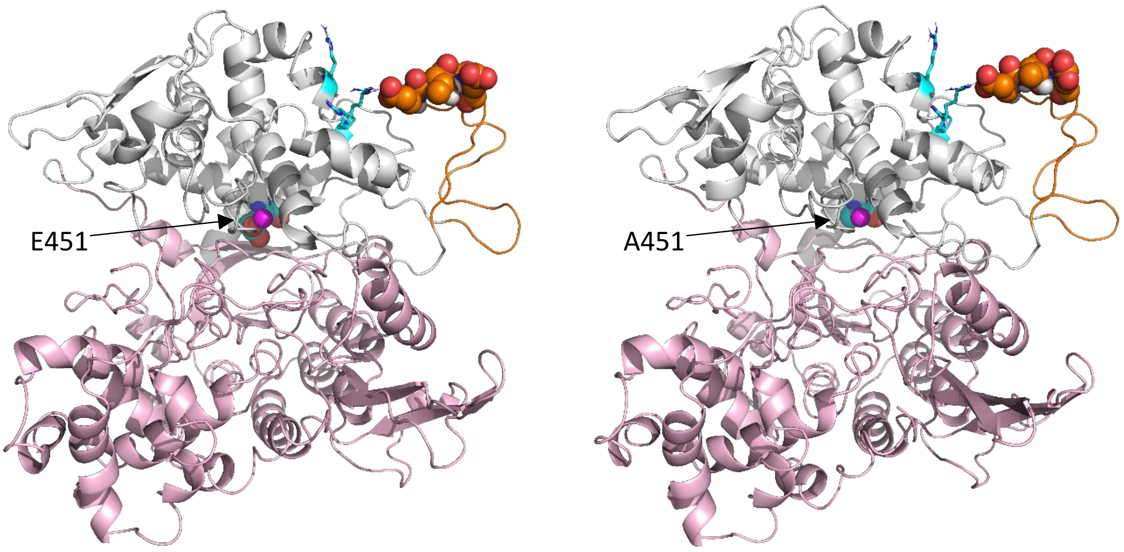

a) b)

Figure S2. The equilibrated structures of: a) WT DPP3 and b) E451A mutant of DPP3 used as starting structures for standard MD and ASMD simulations. The part of ETGE-loop (Q463–Y489) is highlighted in orange, with ETGE motif residues represented as van der Waals spheres. The zinc ion is depicted as a magenta sphere, while the mutated amino acid in its vicinity is also shown as spheres, with carbon atoms colored cyan. Additionally, three arginine residues from the upper protein domain, which form hydrogen bonds with the ETGE motif, are displayed in a licorice representation also with carbon atoms colored cyan. Only polar hydrogen atoms are shown.

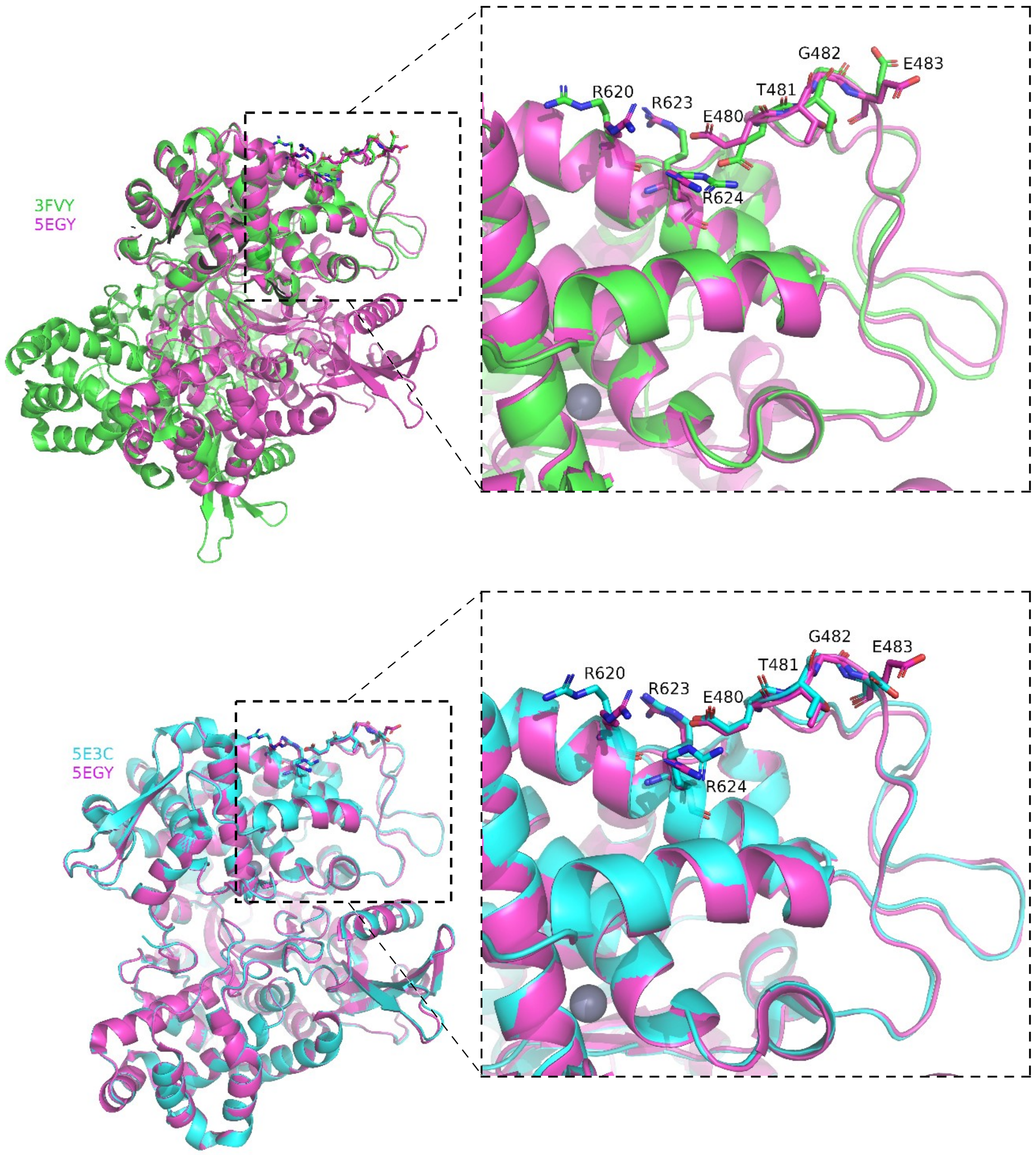

Figure S3. Alignment of the upper domain (K337–T374 and R421–S668) of different DPP3 structures: the WT ligand-free structure in the open conformation (pdb_00003fvy, green), the E451A mutant of ligand-free DPP3 in the closed conformation (pdb_00005egy, magenta), and the E451A mutant of DPP3 in complex with the IVYPW peptide (pdb_00005e3c, cyan), obtained from the Protein Data Bank. The zinc ion is shown as a grey sphere. The ETGE motif and surface-exposed arginine residues in the upper domain that interact with it are shown in licorice representation. The RMSD between the upper domains of WT DPP3 and the E451A mutant is 0.618 Å (upper row), while the RMSD between the E451A mutant and the DPP3–E451A–IVYPW complex is 0.324 Å (lower row). These values indicate that neither the mutation nor ligand binding significantly alters the overall fold of the upper domain or the binding of the ETGE loop.

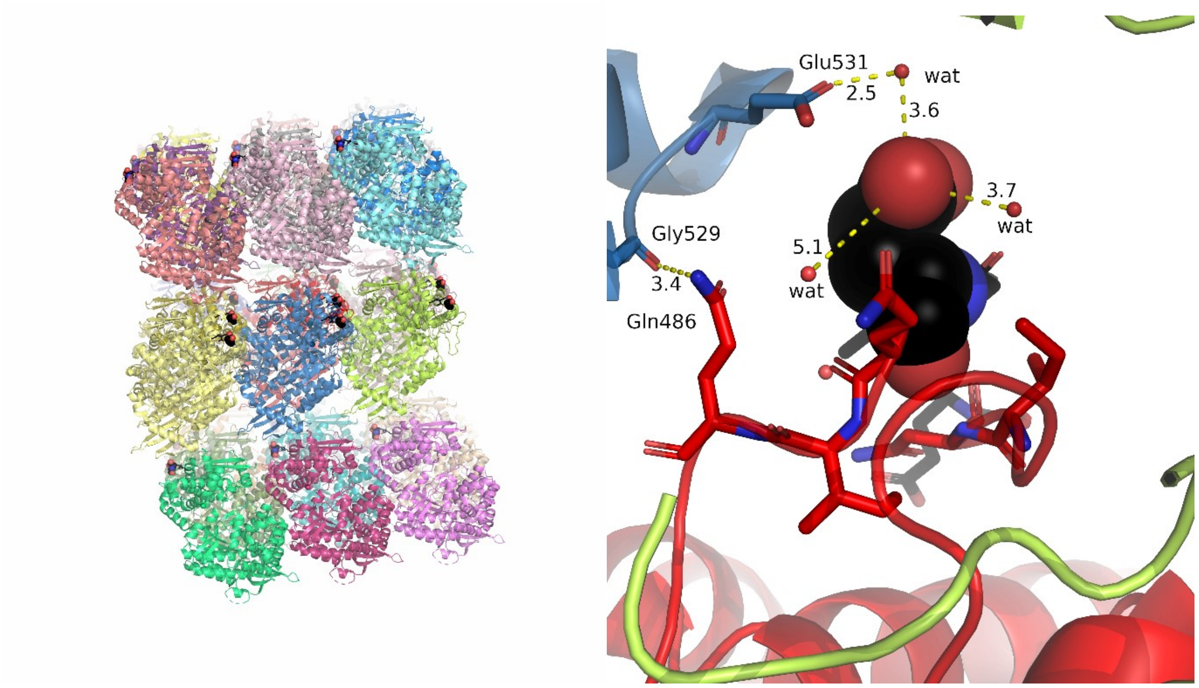

a)

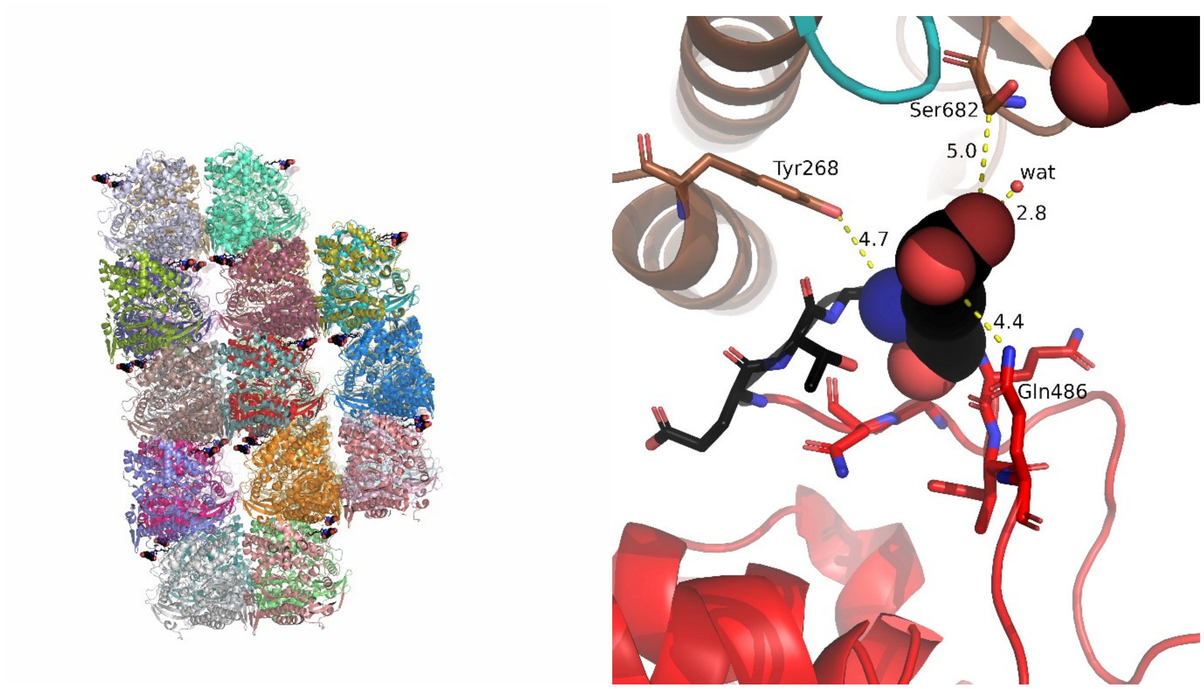

b)

Figure S4. Crystal packing of ligand-free DPP3 structures: a) WT DPP3 (pdb_00003fvy) and b) the E451A mutant (pdb_00005egy). Left panels show the overall crystal packing, while right panels highlight residues located within 5 Å of E486. E486 is shown as spheres with carbon atoms colored black. The neighboring residues are shown in licorice representation and colored according to the symmetry mate to which they belong. Residues constituting the ETGE motif are also shown in licorice representation, with carbon atoms colored black.

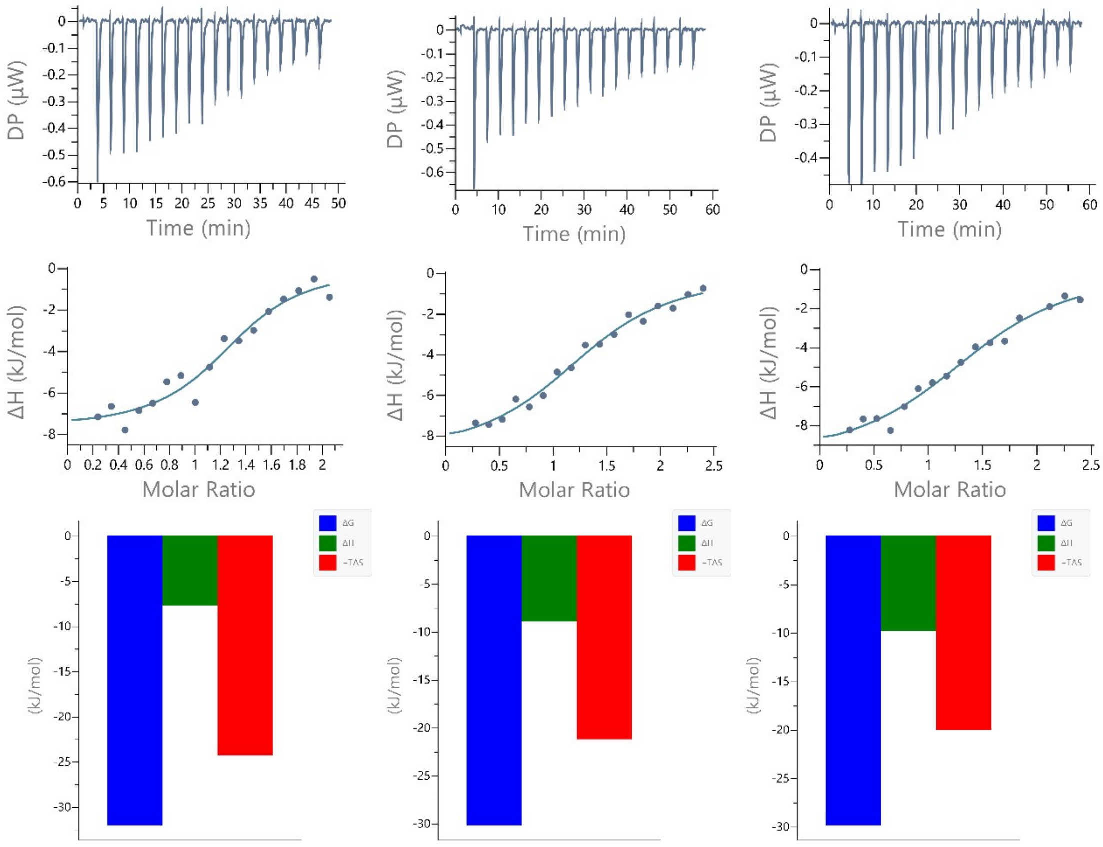

Figure S5. ITC titration and signature plots of WT DPP3 with Kelch domain in 20 mmol dm^{-3} Tris buffer, pH = 8.0 with 150 mmol dm^{-3} NaCl and 1 mmol dm^{-3} TCEP at 25.0 °C. Binding isotherms and data with fitting curves are shown.

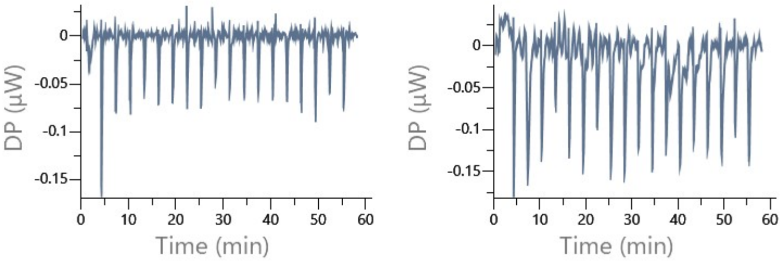

a)

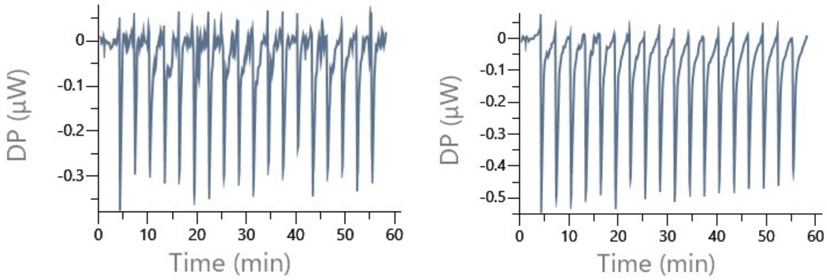

b)

Figure S6. ITC titration of human DPP3-ΔETGE with Kelch in 20 mM Tris buffer, pH = 8.0 with 150 mM NaCl and 1 mM TCEP at 25.0 °C. Binding isotherms and data with fitting curves are shown for: a) direct titrations (Kelch to DPP3-ΔETGE) and b) reverse titrations (DPP3-ΔETGE to Kelch).

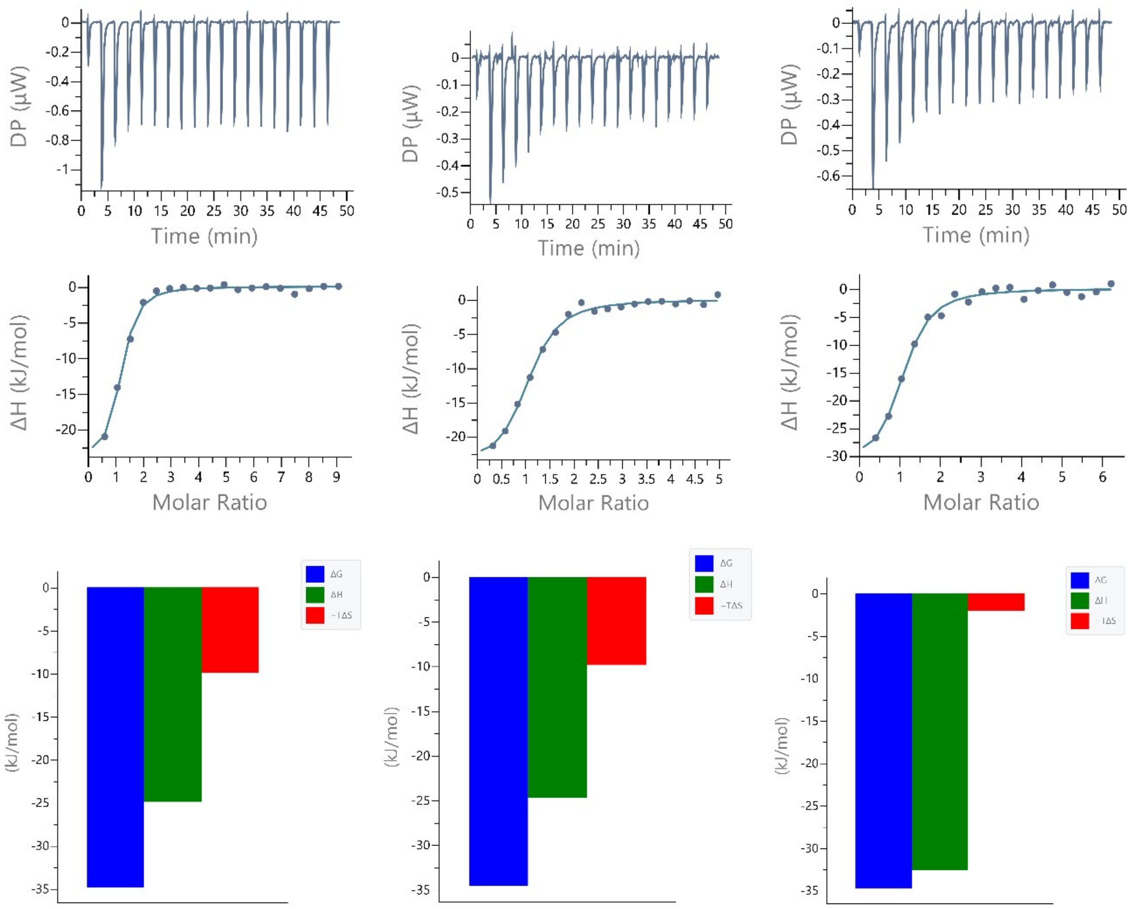

Figure S7. ITC titration and signature plots of E451A mutant of DPP3 with Kelch domain in 20 mmol dm^{-3} Tris buffer, pH = 8.0 with 150 mmol dm^{-3} NaCl and 1 mmol dm^{-3} TCEP at 25.0 °C. Binding isotherms and data with fitting curves are shown.

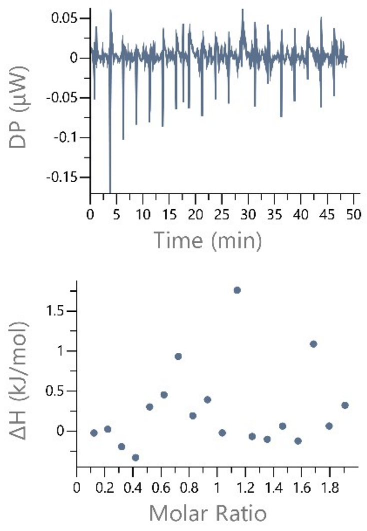

Figure S8. ITC titration of Kelch with IVYPW in 20 mmol dm^{-3} Tris buffer, pH = 8.0 with 150 mmol dm^{-3} NaCl and 1 mmol dm^{-3} TCEP at 25.0 °C.

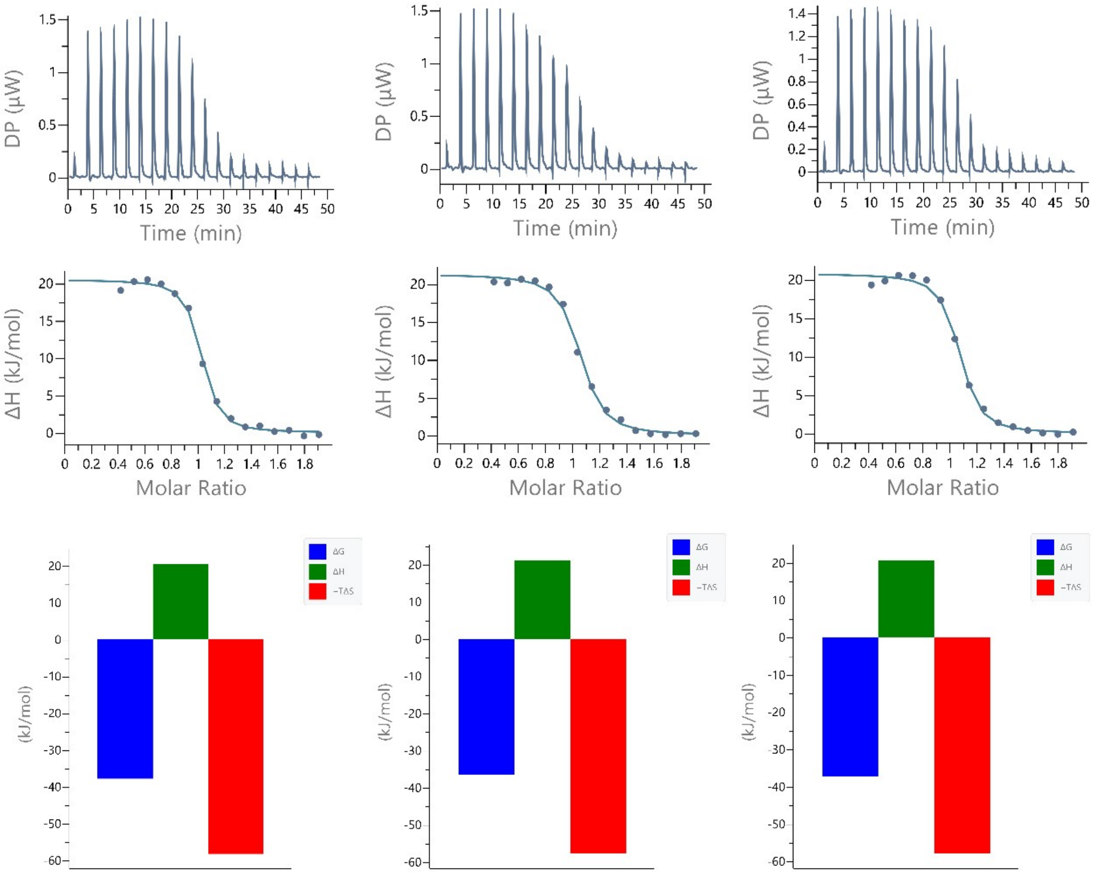

Figure S9. ITC titration of E451A mutant of DPP3 (DPP3-E451A) with IVYPW in 20 mmol dm^{-3} Tris buffer, pH = 8.0 with 150 mmol dm^{-3} NaCl and 1 mmol dm^{-3} TCEP at 25.0 °C.

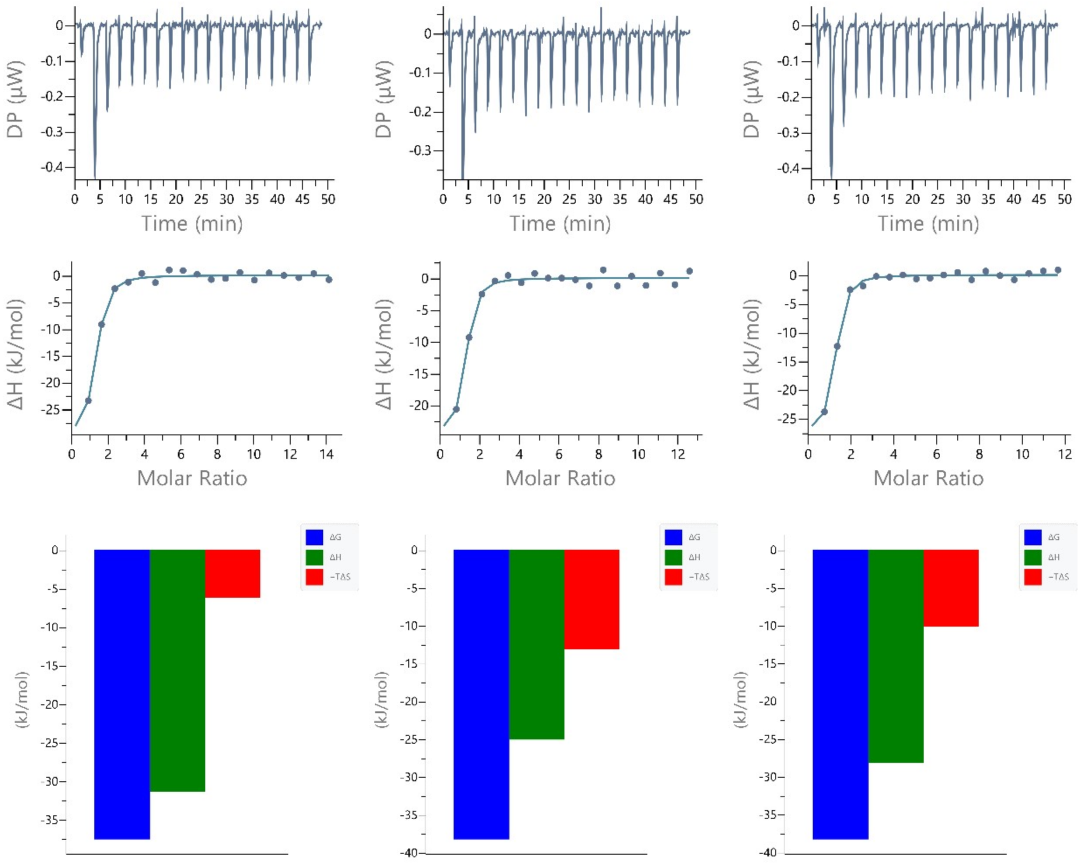

Figure S10. ITC titration and signature plots of DPP3-E451A–IVYPW complex with Kelch domain in 20 mmol dm^{-3} Tris buffer, pH = 8.0 with 150 mmol dm^{-3} NaCl and 1 mmol dm^{-3} TCEP at 25.0 °C. Binding isotherms and data with fitting curves are shown.

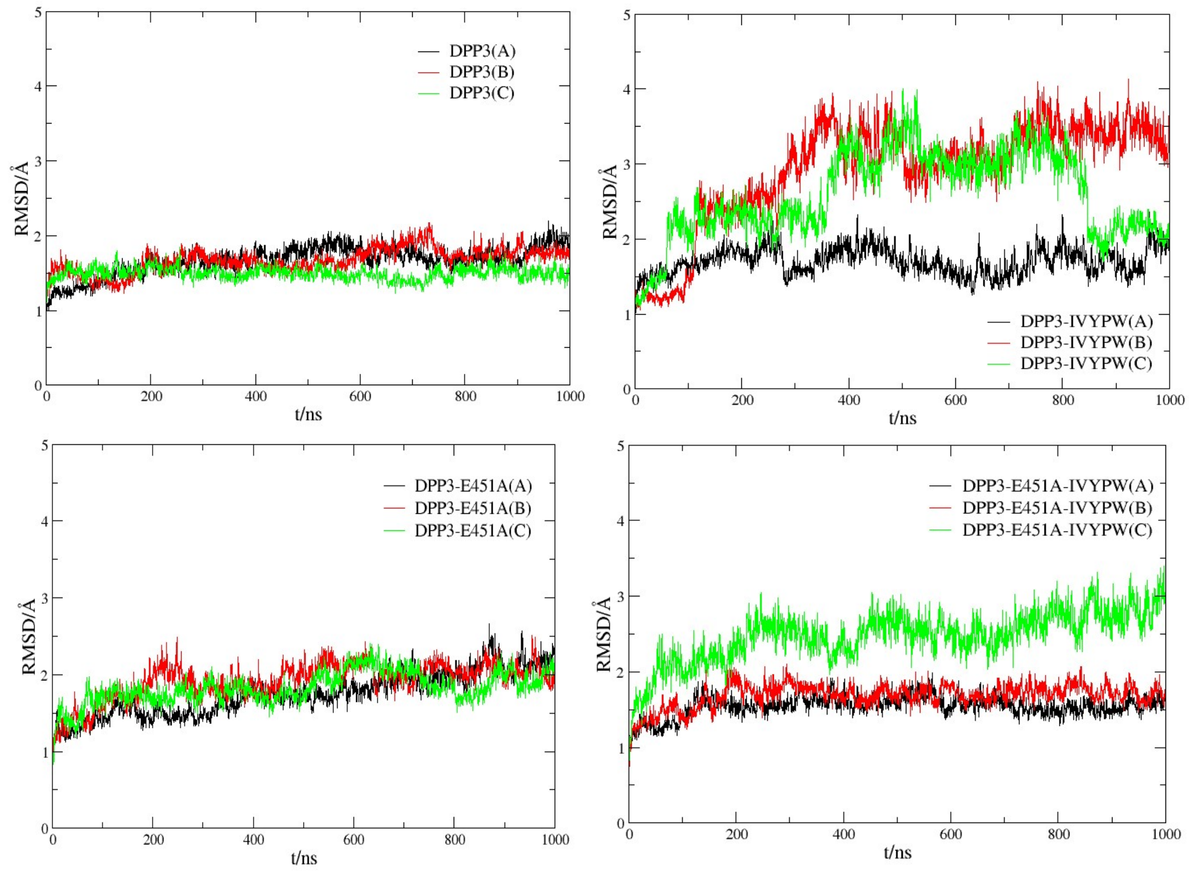

DPP3(A)
DPP3(B)
DPP3(C)
DPP3-IVYPW(A)
DPP3-IVYPW(B)
DPP3-IVYPW(C)
DPP3-E451A(A)
DPP3-E451A(B)
DPP3-E451A(C)
DPP3-E451A-IVYPW(A)
DPP3-E451A-IVYPW(B)
DPP3-E451A-IVYPW(C)
RMSD/Å
t/ns

a)

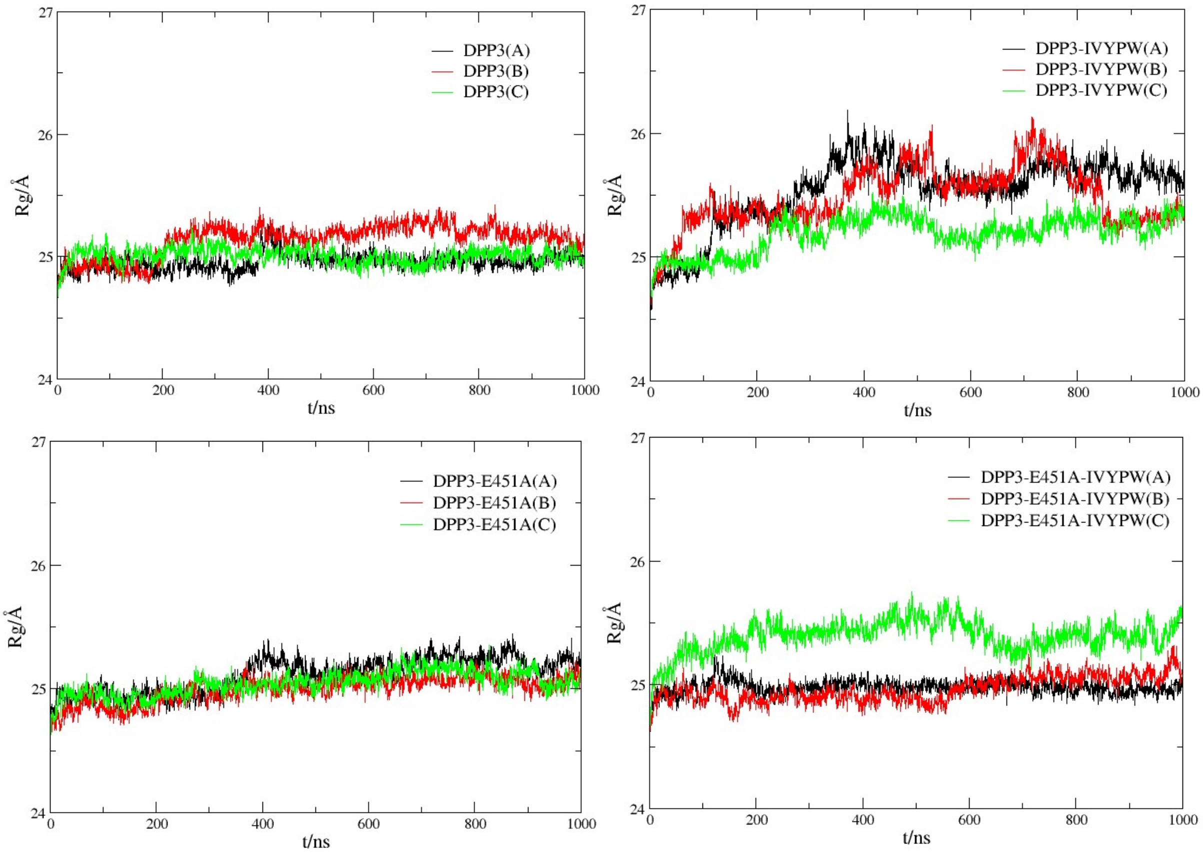

DPP3(A)
DPP3(B)
DPP3(C)
DPP3-IVYPW(A)
DPP3-IVYPW(B)
DPP3-IVYPW(C)
DPP3-E451A(A)
DPP3-E451A(B)
DPP3-E451A(C)
DPP3-E451A-IVYPW(A)
DPP3-E451A-IVYPW(B)
DPP3-E451A-IVYPW(C)
Rg/Å
t/ns

b)

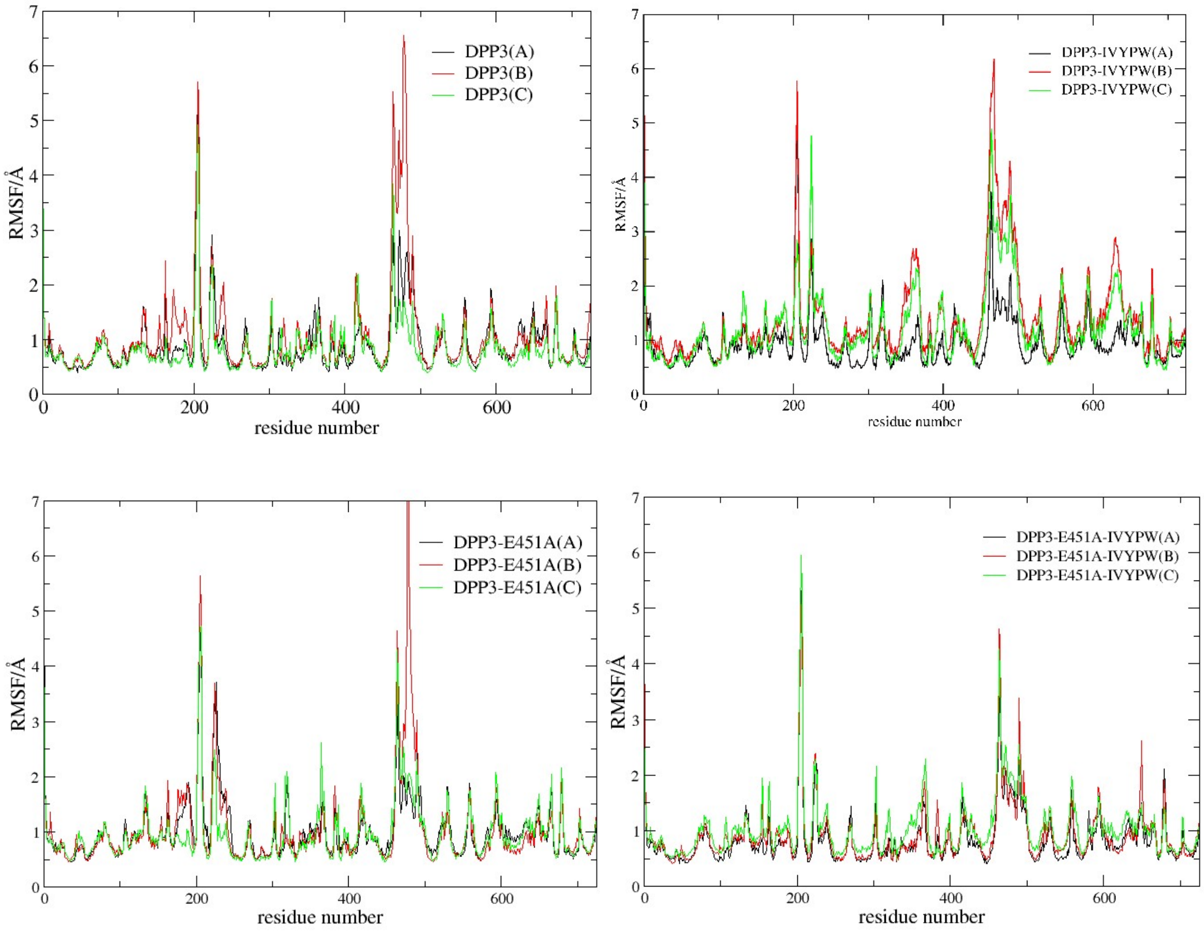

c)

Figure S11. a) RMSD, b) radius of gyration (Rg), and c) RMSF profiles calculated for backbone heavy atoms from three independent 1 µs MD simulations of the following systems: DPP3, DPP3–IVYPW, DPP3-E451A, and DPP3-E451A–IVYPW. Atoms belonging to the flexible loop were excluded from the RMSD and Rg analyses.

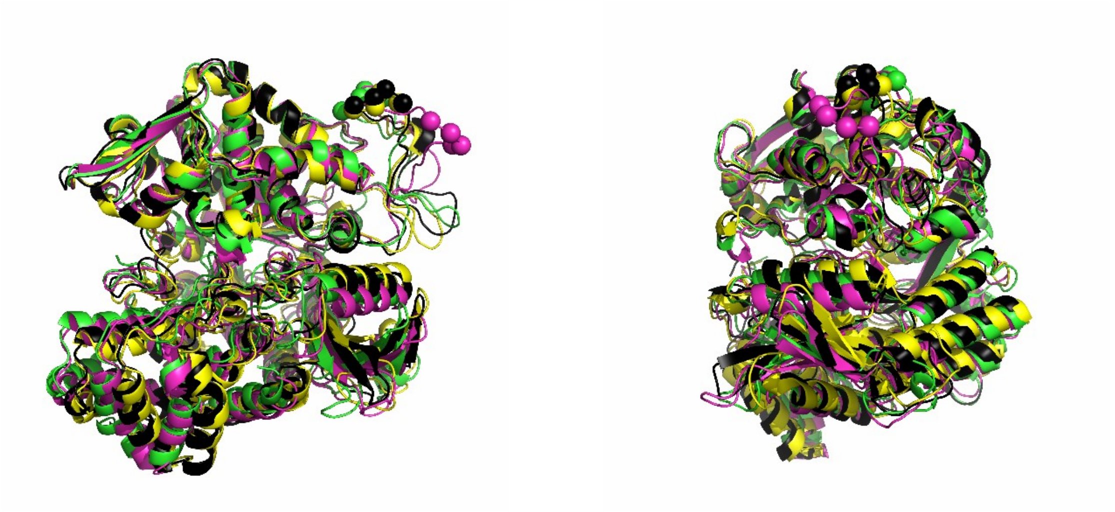

a) **DPP3**

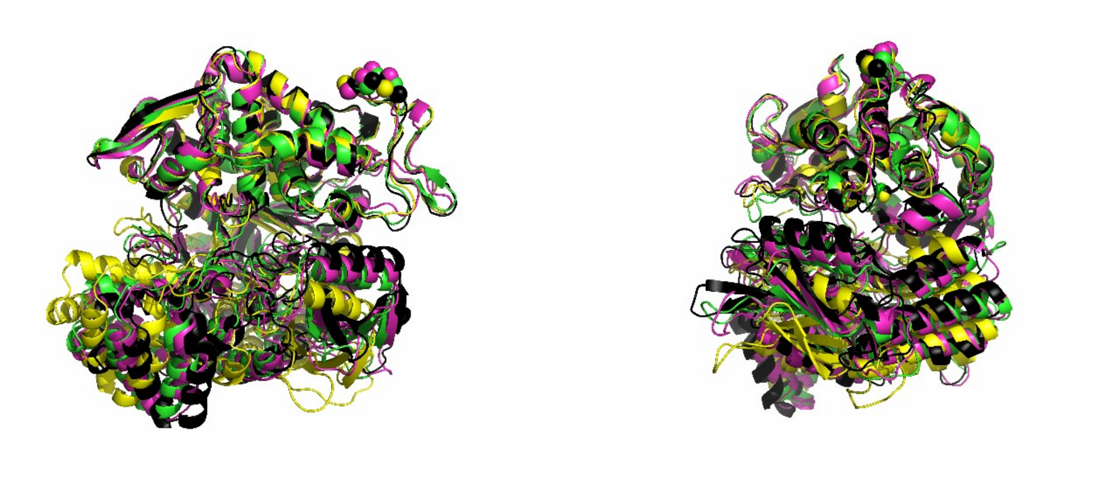

b) **DPP3–IVYPW**

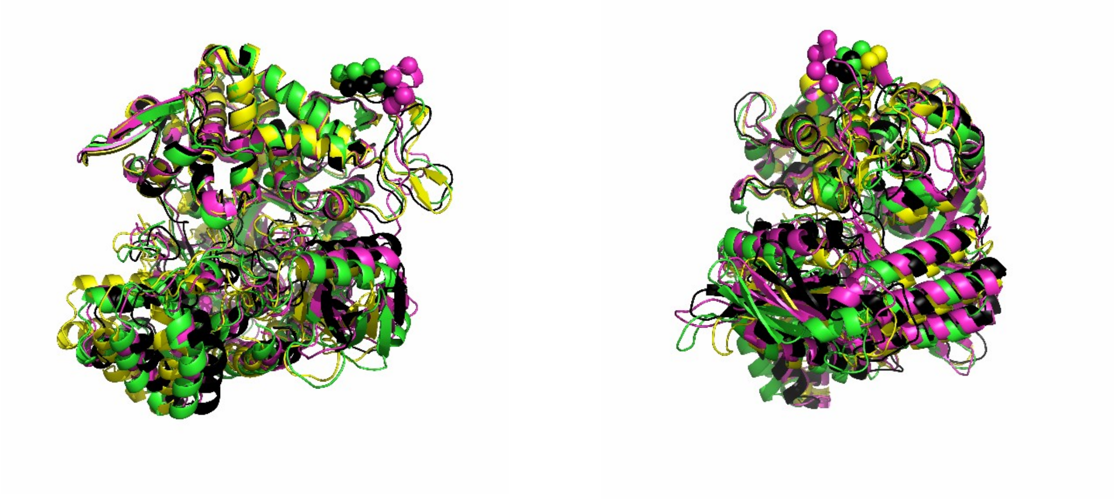

c) **DPP3-E451**

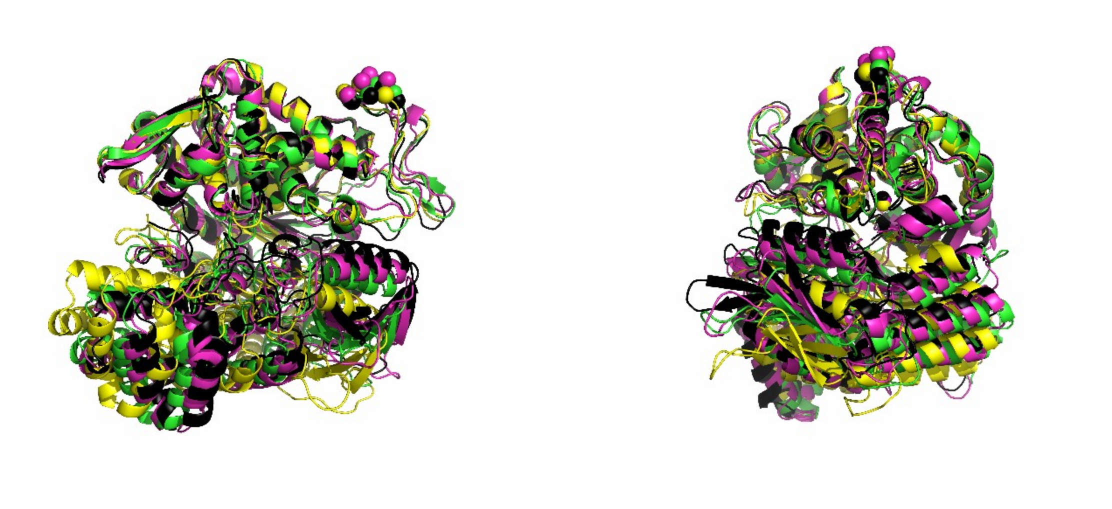

d) **DPP3-E451–IVYPW**

Figure S12. Equilibrated structures (black) used for the productive MD simulations and final structures obtained after three independent 1 μs MD simulations (simulation A, green; simulation B, magenta; simulation C, yellow). Two views are shown; in the right panel, the structure is rotated by 90° relative to the view shown in the left panel. Structures are aligned based on the Cα atoms of the upper domain of the initial structure. The zinc ion and the Cα atoms of the ETGE motif are shown as spheres.

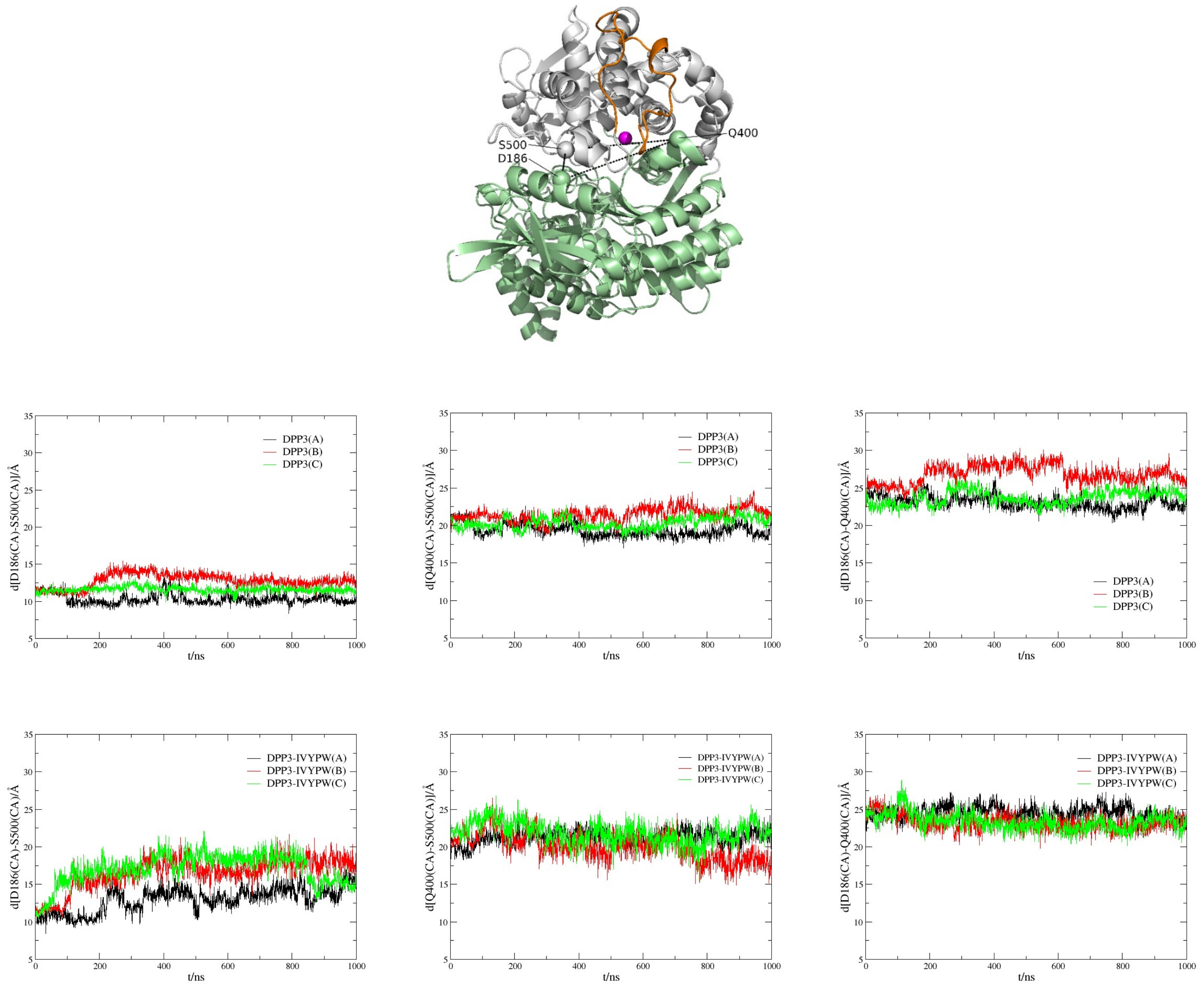
S500
D186
Q400
d[D186(CA)-S500(CA)]/Å
t/ns
DPP3(A)
DPP3(B)
DPP3(C)
d[Q400(CA)-S500(CA)]/Å
t/ns
DPP3(A)
DPP3(B)
DPP3(C)
d[D186(CA)-Q400(CA)]/Å
t/ns
DPP3(A)
DPP3(B)
DPP3(C)
d[D186(CA)-S500(CA)]/Å
t/ns
DPP3-IVYPW(A)
DPP3-IVYPW(B)
DPP3-IVYPW(C)
d[Q400(CA)-S500(CA)]/Å
t/ns
DPP3-IVYPW(A)
DPP3-IVYPW(B)
DPP3-IVYPW(C)
d[D186(CA)-Q400(CA)]/Å
t/ns
DPP3-IVYPW(A)
DPP3-IVYPW(B)
DPP3-IVYPW(C)

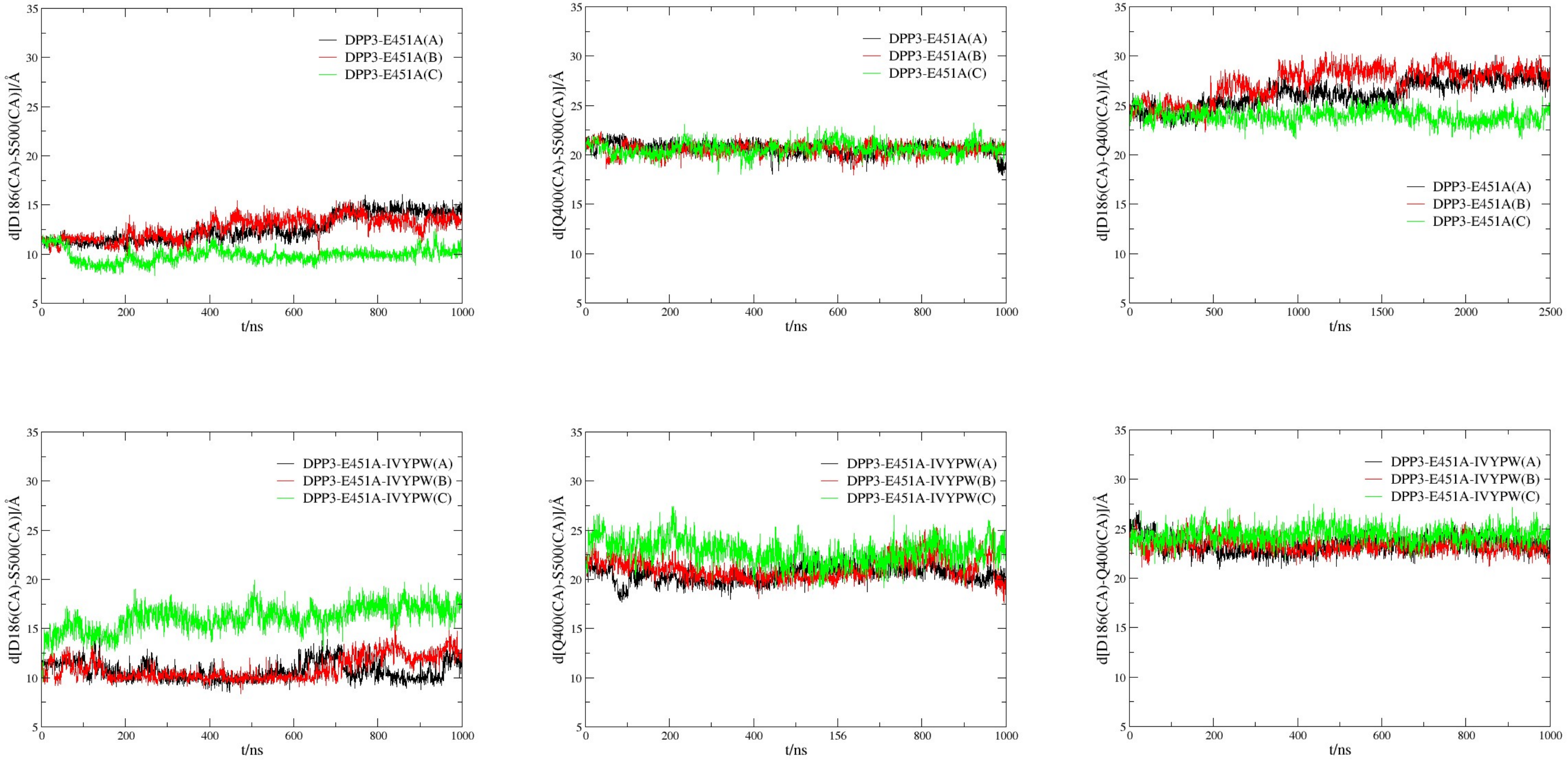

Figure S13. Changes in distances between the α-helices surrounding the ligand entry/exit site, which come into close proximity during the transition of the protein from open to closed conformation, during three independent 1 μs MD simulations of the following systems: DPP3, DPP3–IVYPW, DPP3-E451A, and DPP3-E451A–IVYPW. Distances are represented as changes in Cα–Cα distances between selected residues from the α-helices of the upper domain (colored gray) and the lower domain (colored light green). The corresponding distances are indicated on the DPP3 structure used as the starting point for productive MD simulations as dashed black lines. The zinc cation is shown as a magenta sphere, and the ETGE-loop is colored orange.

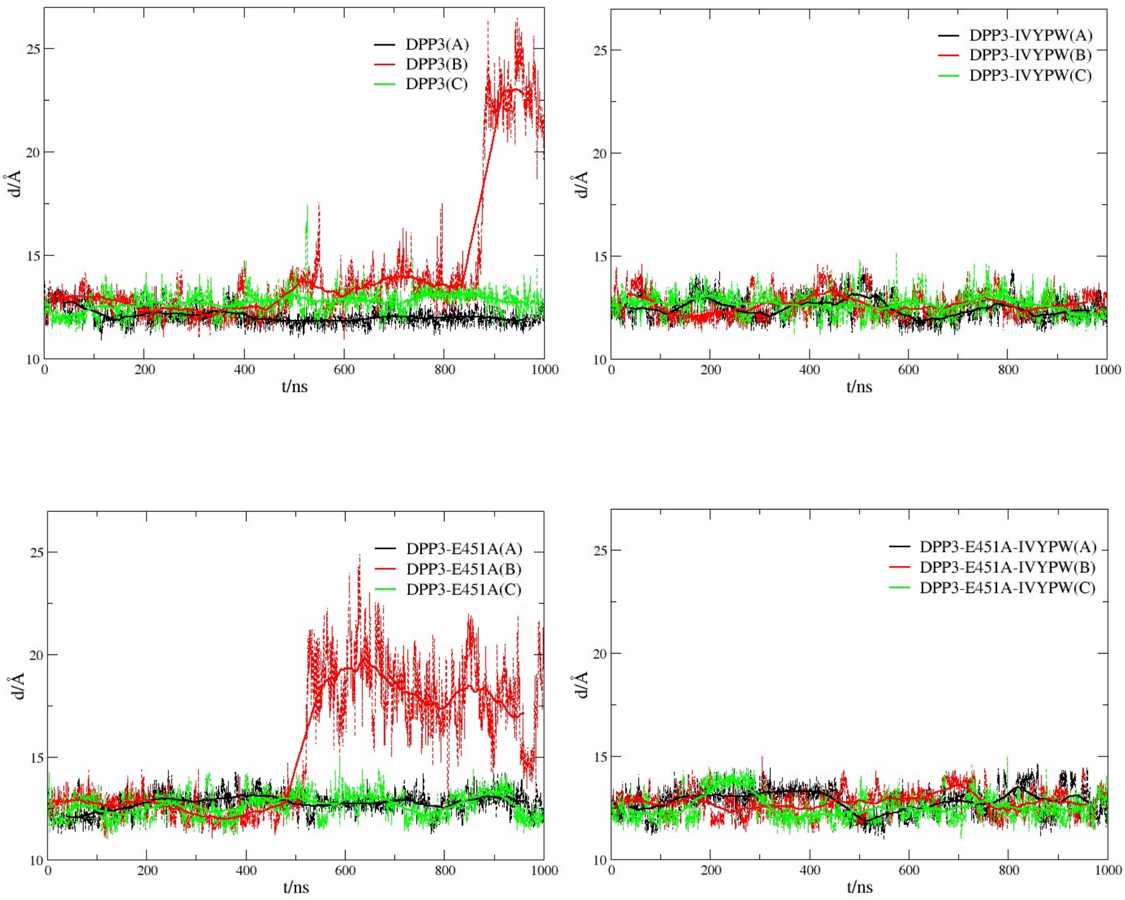

Figure S14. Distance between centers of mass of Cα atoms from the $E^{480}TGE^{483}$ and L622-S630 motifs during three independent 1 µs MD simulations of the following systems: DPP3, DPP3–IVYPW, DPP3-E451A, and DPP3-E451A–IVYPW. Running averages (solid curves) are calculated for subsets of 200 data points.

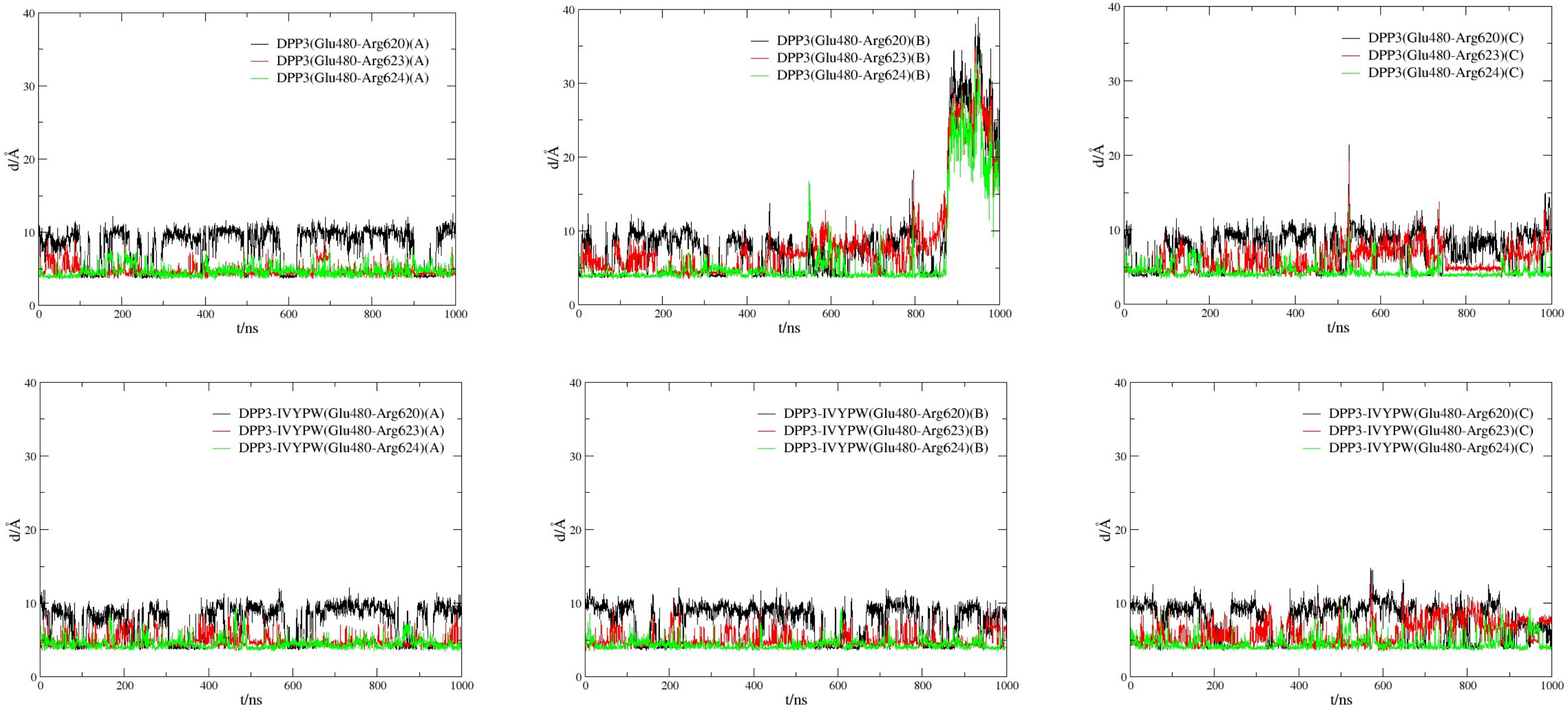

DPP3(Glu480-Arg620)(A)
DPP3(Glu480-Arg623)(A)
DPP3(Glu480-Arg624)(A)
DPP3(Glu480-Arg620)(B)
DPP3(Glu480-Arg623)(B)
DPP3(Glu480-Arg624)(B)
DPP3(Glu480-Arg620)(C)
DPP3(Glu480-Arg623)(C)
DPP3(Glu480-Arg624)(C)
DPP3-IVYPW(Glu480-Arg620)(A)
DPP3-IVYPW(Glu480-Arg623)(A)
DPP3-IVYPW(Glu480-Arg624)(A)
DPP3-IVYPW(Glu480-Arg620)(B)
DPP3-IVYPW(Glu480-Arg623)(B)
DPP3-IVYPW(Glu480-Arg624)(B)
DPP3-IVYPW(Glu480-Arg620)(C)
DPP3-IVYPW(Glu480-Arg623)(C)
DPP3-IVYPW(Glu480-Arg624)(C)
d/Å
t/ns
0
10
20
30
40
200
400
600
800
1000

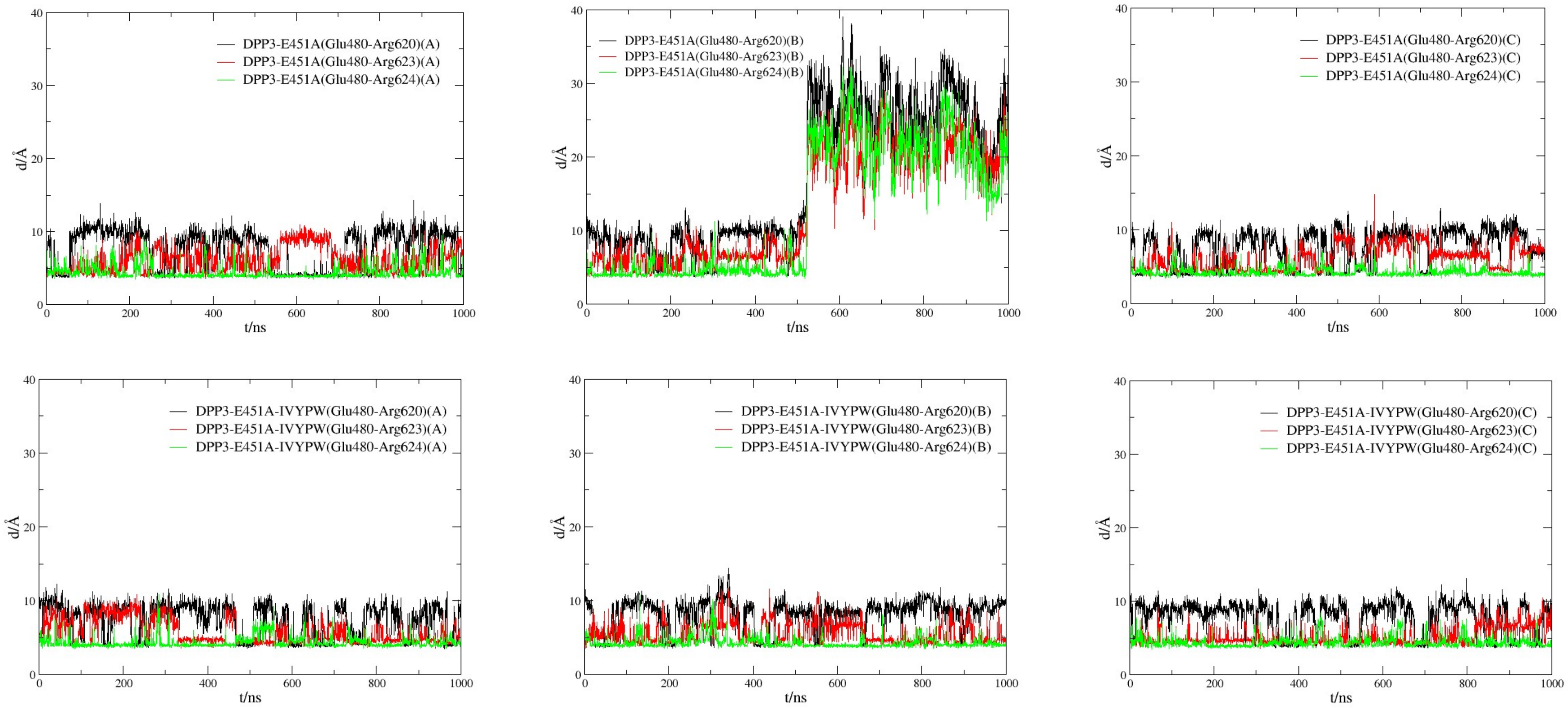

Figure S15. Distances between the carboxyl carbon of Glu480 and the guanidino carbon of Arg620, Arg623, and Arg624 during three independent 1 µs MD simulations of the following systems: DPP3, DPP3–IVYPW, DPP3-E451A, and DPP3-E451A–IVYPW.

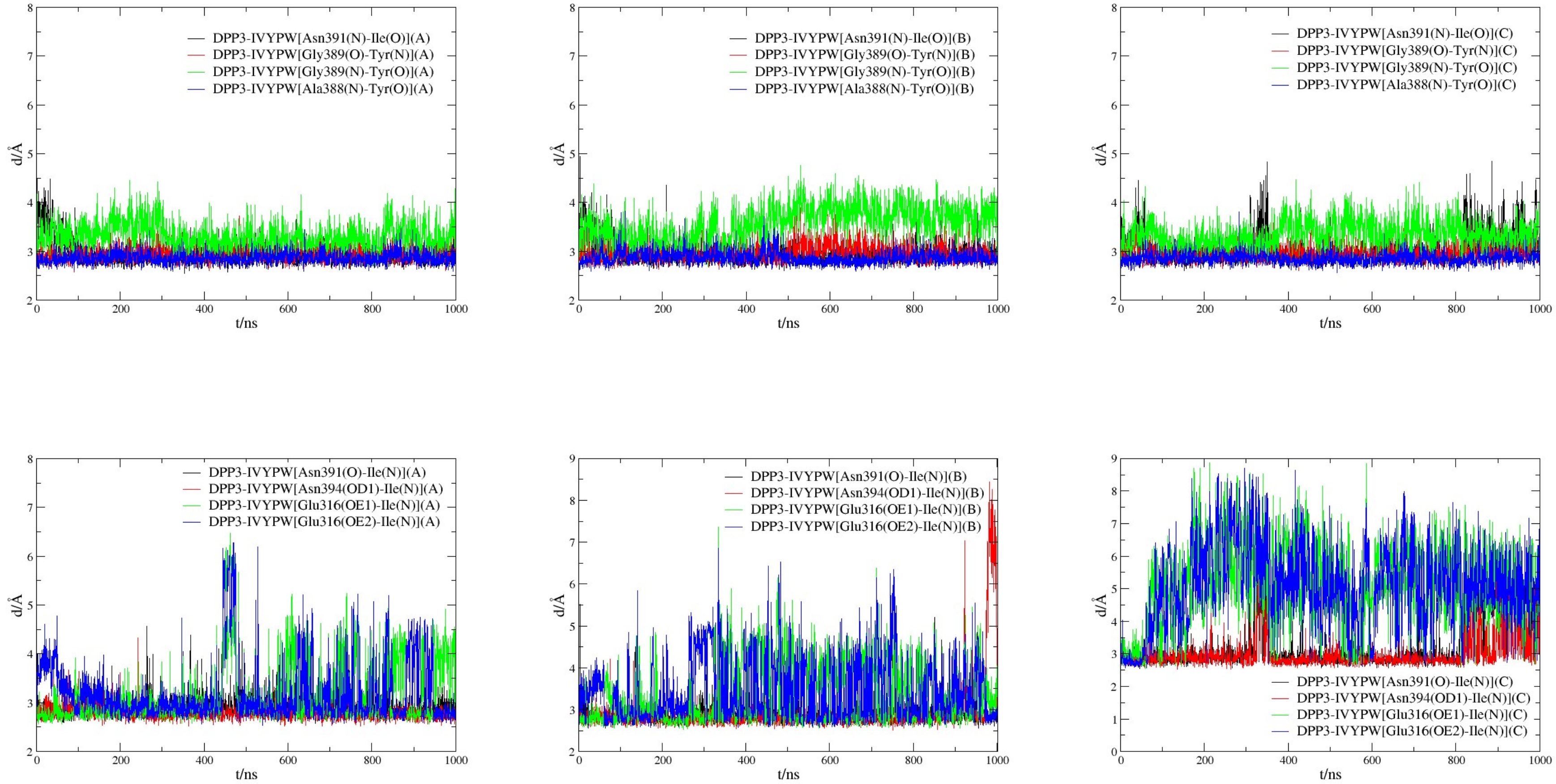
DPP3-IVYPW[Asn391(N)-Ile(O)](A)
DPP3-IVYPW[Gly389(O)-Tyr(N)](A)
DPP3-IVYPW[Gly389(N)-Tyr(O)](A)
DPP3-IVYPW[Ala388(N)-Tyr(O)](A)
DPP3-IVYPW[Asn391(N)-Ile(O)](B)
DPP3-IVYPW[Gly389(O)-Tyr(N)](B)
DPP3-IVYPW[Gly389(N)-Tyr(O)](B)
DPP3-IVYPW[Ala388(N)-Tyr(O)](B)
DPP3-IVYPW[Asn391(N)-Ile(O)](C)
DPP3-IVYPW[Gly389(O)-Tyr(N)](C)
DPP3-IVYPW[Gly389(N)-Tyr(O)](C)
DPP3-IVYPW[Ala388(N)-Tyr(O)](C)
DPP3-IVYPW[Asn391(O)-Ile(N)](A)
DPP3-IVYPW[Asn394(OD1)-Ile(N)](A)
DPP3-IVYPW[Glu316(OE1)-Ile(N)](A)
DPP3-IVYPW[Glu316(OE2)-Ile(N)](A)
DPP3-IVYPW[Asn391(O)-Ile(N)](B)
DPP3-IVYPW[Asn394(OD1)-Ile(N)](B)
DPP3-IVYPW[Glu316(OE1)-Ile(N)](B)
DPP3-IVYPW[Glu316(OE2)-Ile(N)](B)
DPP3-IVYPW[Asn391(O)-Ile(N)](C)
DPP3-IVYPW[Asn394(OD1)-Ile(N)](C)
DPP3-IVYPW[Glu316(OE1)-Ile(N)](C)
DPP3-IVYPW[Glu316(OE2)-Ile(N)](C)
d/Å
t/ns

a)

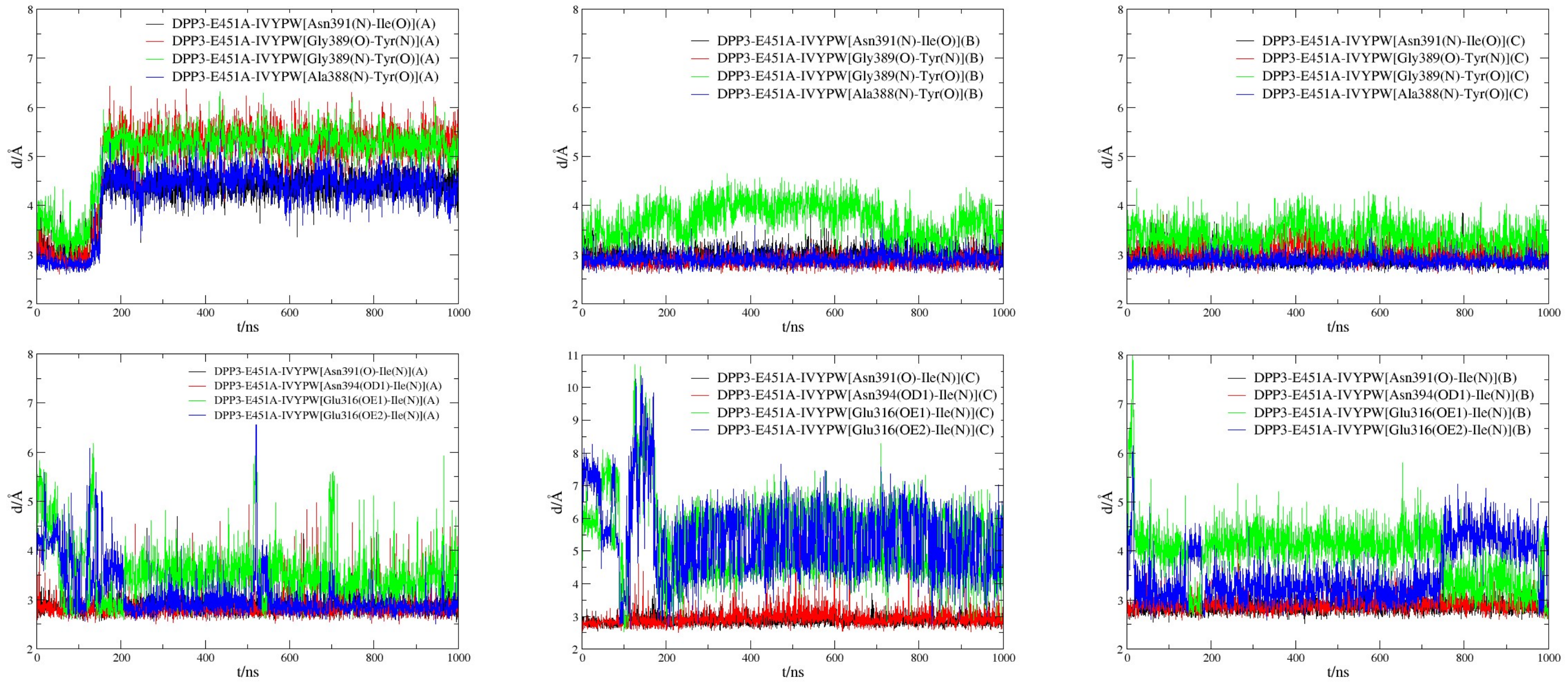

b)

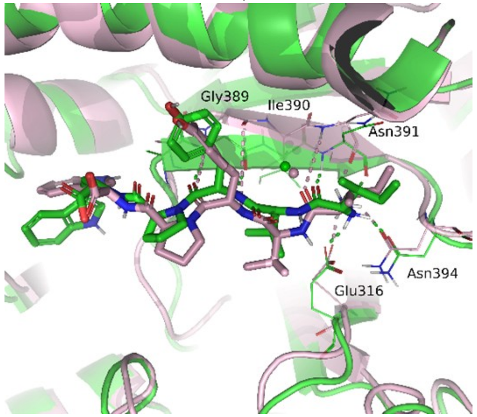

c)

Figure S16. Change in selected distances that define binding of the ligand (IVYPW) in a form of a β-sheet in antiparallel fashion to a β-sheet from a lower protein domain during MD simulations of the complexes: a) WT and b) E451A variant. C) Structure shows binding of the ligand in the enzyme binding site as obtained after 1 µs of MD simulations in the complexes: DPP3–IVYPW (green) and DPP3-E451A–IVYPW (pink), simulation A. Zinc ion is shown as sphere. Structures are aligned according to Cα atoms. Dashed lines represent selected distances.

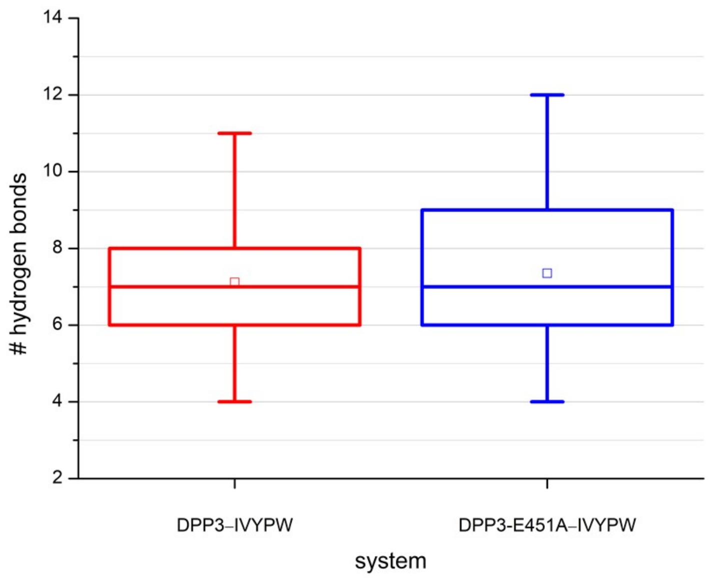

Figure S17. Box plots showing the number of hydrogen bonds between the ligand (IVYPW) and protein during all three independent 1 µs MD simulations of complexes. Data were extracted from structures sampled every 400 ps over 1-µs MD simulations. A hydrogen bond was defined as having a donor-acceptor distance < 3.2 Å and a donor-hydrogen-acceptor angle < 40°.

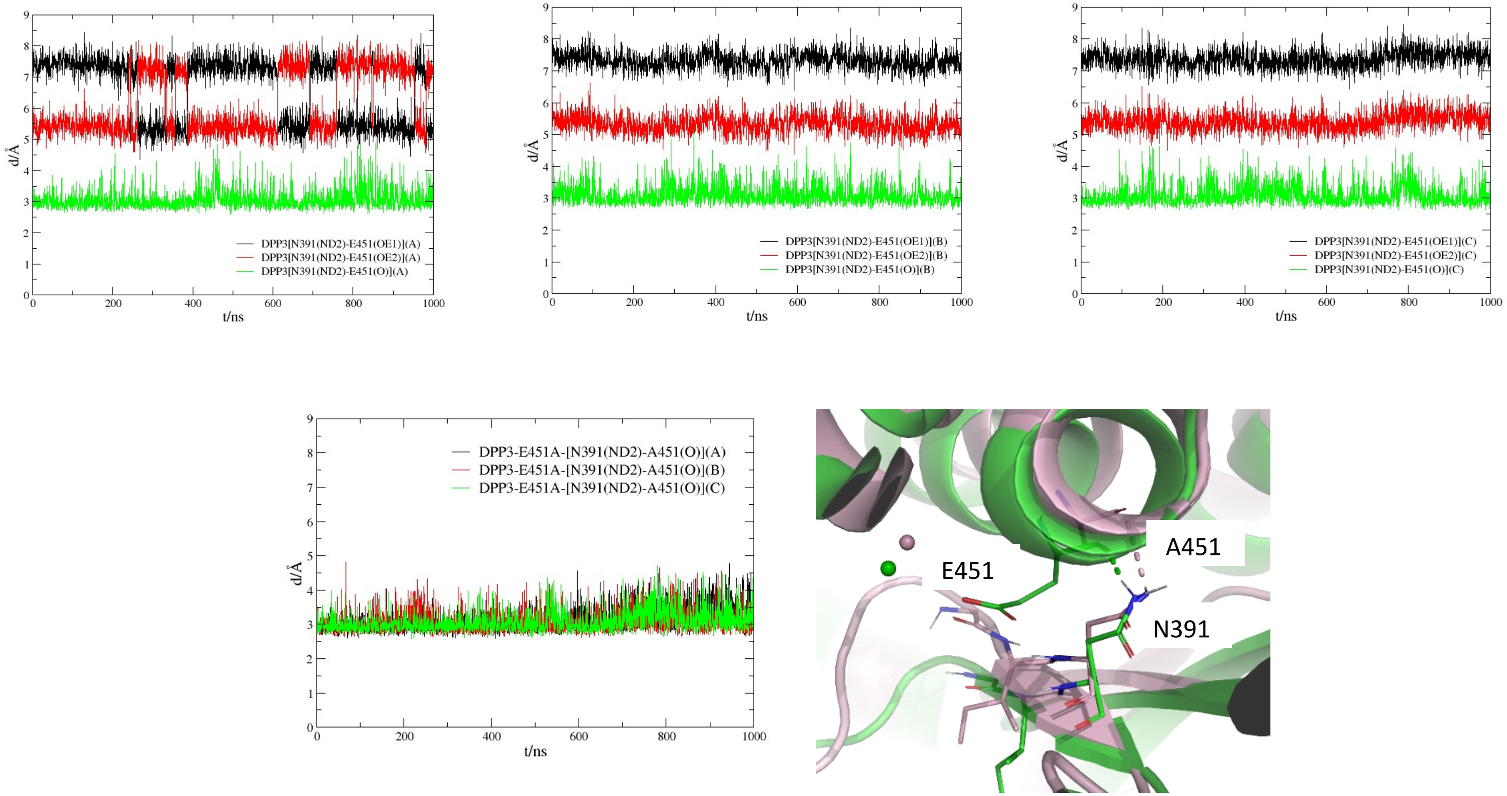
DPP3[N391(ND2)-E451(OE1)](A)
DPP3[N391(ND2)-E451(OE2)](A)
DPP3[N391(ND2)-E451(O)](A)
DPP3[N391(ND2)-E451(OE1)](B)
DPP3[N391(ND2)-E451(OE2)](B)
DPP3[N391(ND2)-E451(O)](B)
DPP3[N391(ND2)-E451(OE1)](C)
DPP3[N391(ND2)-E451(OE2)](C)
DPP3[N391(ND2)-E451(O)](C)
DPP3-E451A-[N391(ND2)-A451(O)](A)
DPP3-E451A-[N391(ND2)-A451(O)](B)
DPP3-E451A-[N391(ND2)-A451(O)](C)
d/Å
t/ns
A451
E451
N391

a)

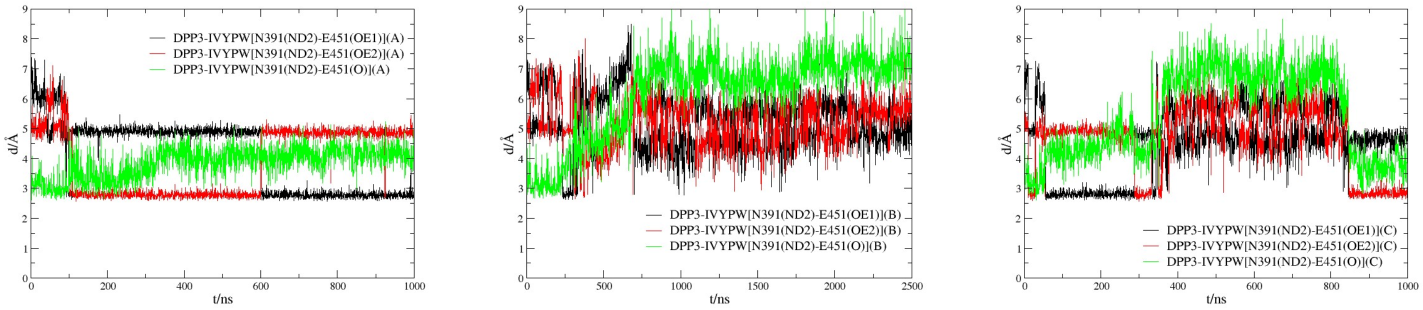
DPP3-IVYPW[N391(ND2)-E451(OE1)](A)
DPP3-IVYPW[N391(ND2)-E451(OE2)](A)
DPP3-IVYPW[N391(ND2)-E451(O)](A)
DPP3-IVYPW[N391(ND2)-E451(OE1)](B)
DPP3-IVYPW[N391(ND2)-E451(OE2)](B)
DPP3-IVYPW[N391(ND2)-E451(O)](B)
DPP3-IVYPW[N391(ND2)-E451(OE1)](C)
DPP3-IVYPW[N391(ND2)-E451(OE2)](C)
DPP3-IVYPW[N391(ND2)-E451(O)](C)
d/Å
t/ns

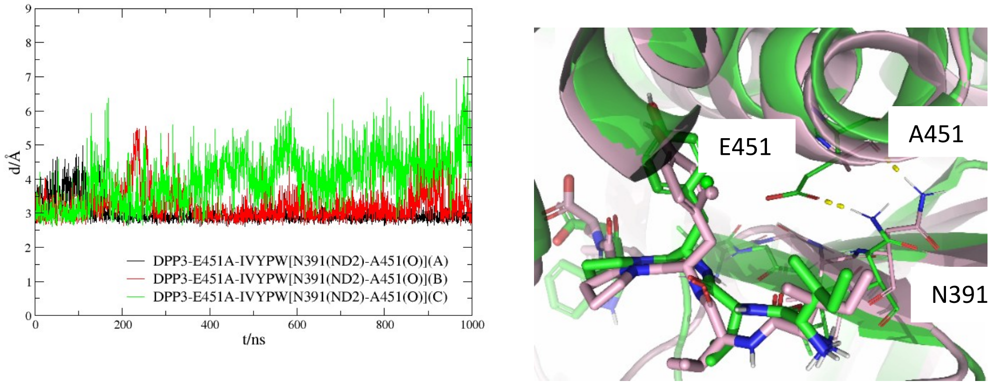

b)

Figure S18. Distances between selected atoms of the Asn391 from the lower protein domain and Glu451 or Ala451 from the upper protein domain during three independent 1 μs MD simulations of WT (zinc, protein and carbon atoms colored green) and E451A (zinc, protein and carbon atoms colored pink) DPP3 in: a) ligand-free system and b) in complex with IVYPW (shown with thicker sticks).

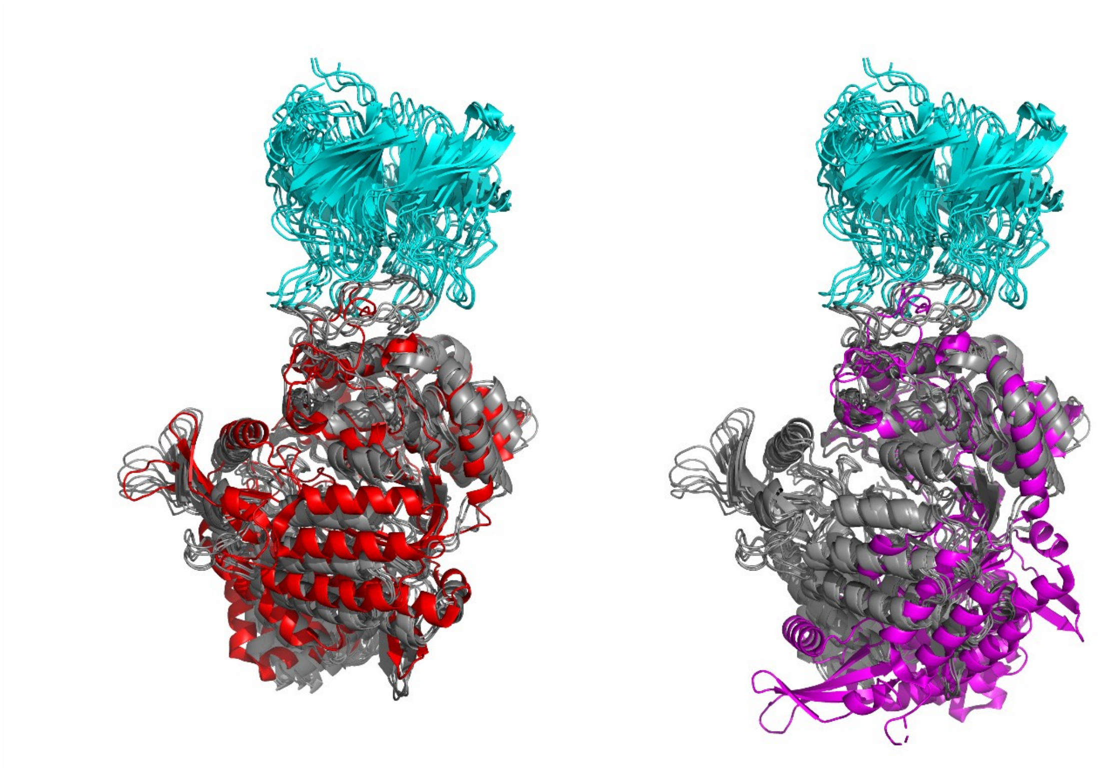

a) b)

Figure S19. Alignment of DPP3 structures from the complexes used in the SAXS analysis by Matić et al. (*J. Biomol. Struct. Dyn.* 2021 39(18): 6870–6881) (DPP3 in gray, Kelch domain in cyan) with: a) the closed structure of the enzyme (PDB ID: 5EGY, shown in red) and b) the open structure (PDB ID: 3FVY, shown in magenta) obtained from the PDB.

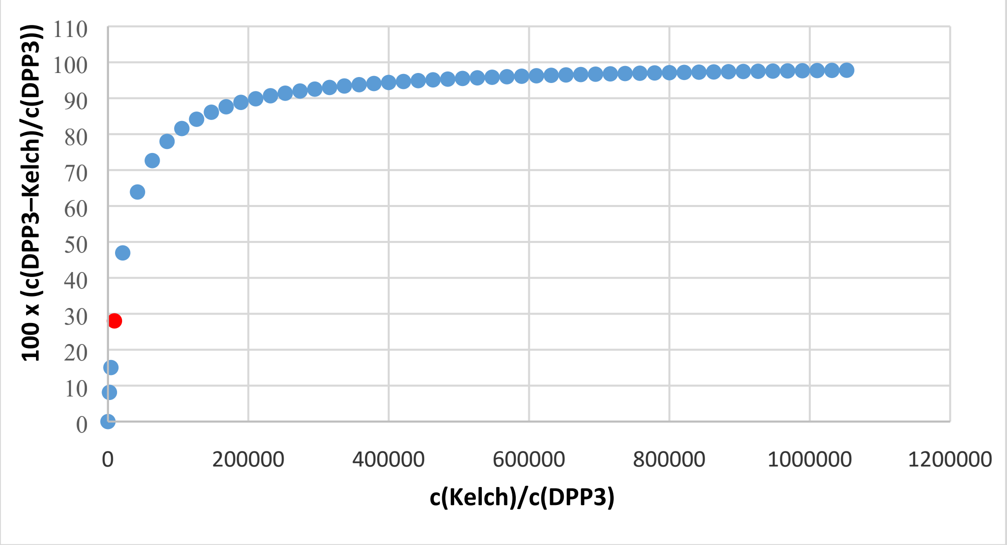

Figure S20. Percentage of DPP3–Kelch complex formed in solution prepared by mixing 5 μL of 0.116 μmol dm^{-3} DPP3 with 12.5 μL of Kelch at varying concentrations in 3 mL of 0.5 mol dm^{-3} Tris buffer (pH 7.4). The equilibrium complex concentration was calculated using the association constant ($K_a = 1/K_d$) obtained from the direct ITC measurements performed in this study. The value highlighted in red indicates the percentage of DPP3–Kelch complex formed (28%) when 12.5 μL of 428 μmol dm^{-3} Kelch was used.

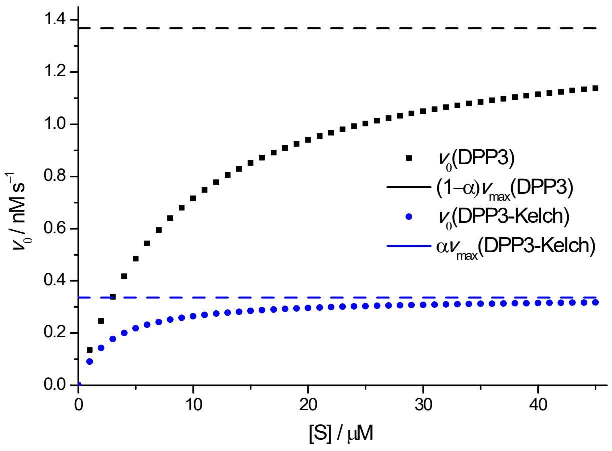

Figure S21. Simulation of kinetic data for Arg_2-2NA hydrolysis by a mixture of free DPP3 and the DPP3–Kelch complex, using the experimentally determined kinetic parameters of both enzyme forms.

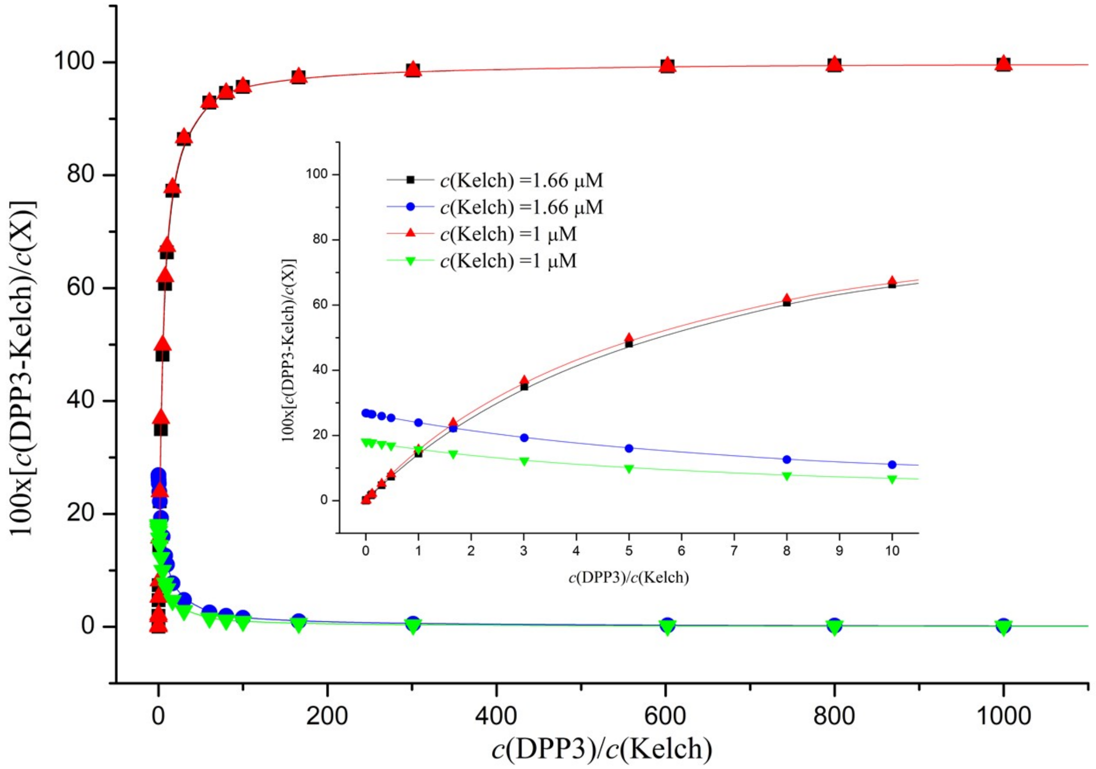

Figure S22. Fraction of Kelch sites bound by DPP3 (X=Kelch; back and red symbols and lines) and fraction of DPP3 in complex with Kelch (X=DPP3, blue and green symbols and lines) as a function of the DPP3:Kelch molar ratio. The concentration of available Kelch binding sites was fixed at 1.66 μM (black squares and blue circles) and 1 μM (red up-pointing triangles and green down-pointing triangles), corresponding to the estimated number of Kelch binding sites accessible for DPP3 binding under basal and oxidative conditions, respectively, assuming a cellular Keap1 concentration of ~1 μM. The DPP3 concentration was varied. The inset graph provides an enlarged view of the region of the main graph corresponding to low *c*(DPP3)/*c*(Kelch) molar ratios.

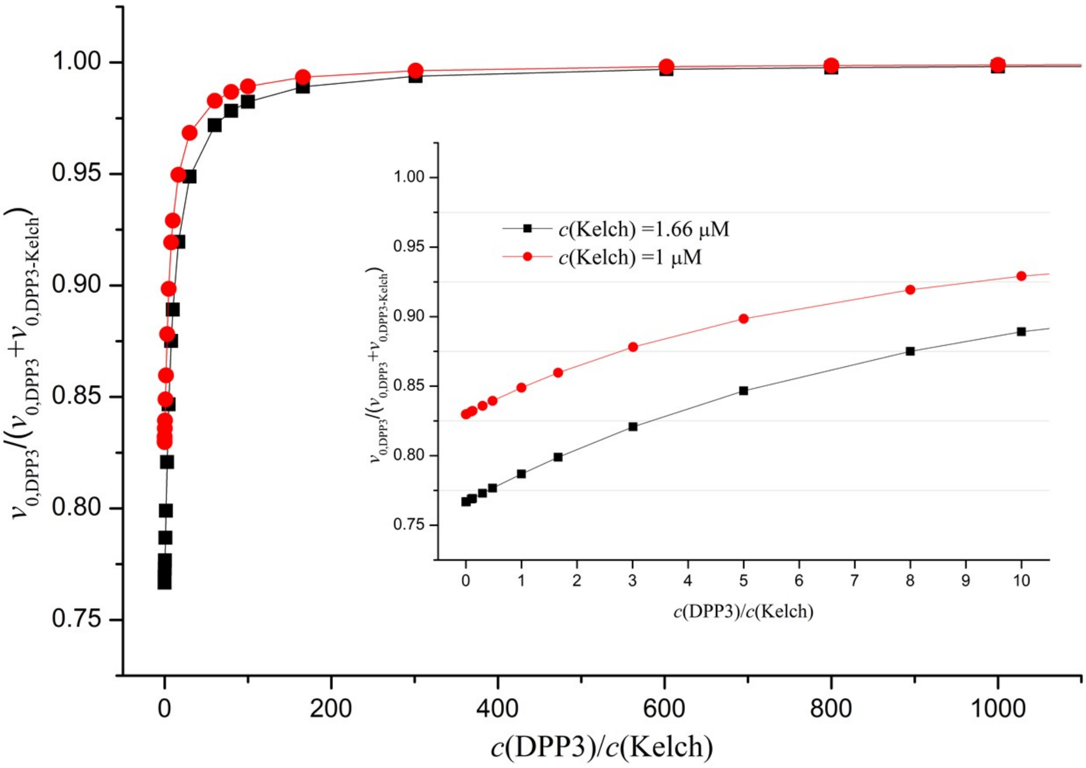

Figure S23. The ratio of hydrolysis rates between DPP3 ($v_{0,DPP3}$) and DPP3 partially bound in a complex with Kelch in addition to the free DPP3 ($v_{0,DPP3} + v_{0,DPP3\text{-}Kelch}$) is shown as a function of DPP3:Kelch molar ratio. Hydrolysis rates were calculated using $v_0=(k_{cat}/K_m)[DPP3]_{tot}$, the experimentally determined kinetic parameters of both enzyme forms, and the concentrations of available Kelch biding sites under two conditions: 1.66 μM in the basal state (black squares), and 1 μM under oxidative stress (red circles). The fraction of DPP3 bound to Kelch was computed using a dissociation constant K_d=4.52 μM. The inset graph provides an enlarged view of the region of the main graph corresponding to low *c*(DPP3)/*c*(Kelch) molar ratios.
